# From Chemoenzymatic Synthesis to Biological Effects: Enantiomerically Enriched Vanillin-Derived δ-Iodo-γ-Lactones as Modulators of Cancer Cell Viability and Inducers of Cell Death

**DOI:** 10.3390/ijms27177928

**Published:** 2026-09-05

**Authors:** Anna Dunal, Dominik Poradowski, Stefano Serra, Aleksander Chrószcz, Hanna Pruchnik, Witold Gładkowski

**Affiliations:** 1 Department of Food Chemistry and Biocatalysis, Wrocław University of Environmental and Life Sciences, Norwida 25, 50-375 Wrocław, Poland; 2 Department of Biostructure and Animal Physiology, Division of Animal Anatomy, Faculty of Veterinary Medicine, Wrocław University of Environmental and Life Sciences, Kożuchowska 1, 51-631 Wrocław, Poland; dominik.poradowski@upwr.edu.pl (D.P.); aleksander.chroszcz@upwr.edu.pl (A.C.); 3 Consiglio Nazionale delle Ricerche (C.N.R.), Istituto di Scienze e Tecnologie Chimiche, Via Mancinelli 7, 20131 Milano, Italy; stefano.serra@cnr.it; 4 Department of Physics and Biophysics, Faculty of Biotechnology and Food Sciences, Wrocław University of Environmental and Life Sciences, Norwida 25, 50-375 Wrocław, Poland; hanna.pruchnik@upwr.edu.pl

**Keywords:** enantiomers, iodolactones, vanillin, cell viability, TUNEL assay, DNA fragmentation, cell cycle

## Abstract

Enantiomerically enriched vanillin-derived *cis*- and *trans*-β-aryl-δ-iodo-γ-lactones were synthesized using a chemoenzymatic pathway based on *Candida antarctica* lipase B (CALB)-catalyzed transesterification of racemic (*E*)-4-(4′-benzyloxy-3′-methoxyphenyl)but-3-en-2-ol, followed by Claisen rearrangement, hydrolysis, iodolactonization, and deprotection. The *trans* orientation of substituents on the γ-lactone ring of the target compounds was identified as the key structural feature enhancing cell-viability-reducing activity against selected human cancer cell lines. No statistically significant differences were observed between the enantiomerically enriched pairs of the studied lactones: the most active *trans*-δ-iodo-γ-lactones, **7b**, exhibited similarly high potency (IC_50_ = 3.98 ± 2.60 μM and 5.08 ± 1.30 μM) against the multidrug-resistant (MDR) gastric adenocarcinoma cell line EPG85-257RDB. Their IC_50_ values were not significantly different from that determined for doxorubicin under the same experimental conditions (IC_50_ = 5.48 ± 0.75 μM). Notably, both enantiomers exhibited high selectivity indices (SI = 9.22 and 10.19, respectively). TUNEL analysis demonstrated that enantiomer (4*R*,5*S*,6*R*)-**7b** induced concentration-dependent DNA fragmentation, increasing the percentage of TUNEL-positive cells from 8.30 ± 0.95% to 74.10 ± 4.26%. Flow cytometric analysis further revealed the pronounced accumulation of cells in the G2/M phase, with up to 55.25 ± 2.74% of cells detected at the highest tested concentration. The observed activity of enantiomerically enriched vanillin-derived *trans*-β-aryl-δ-iodo-γ-lactone **7b** in the multidrug-resistant EPG85-257RDB cell line warrants its further investigation in additional drug-resistant cancer models.

## 1. Introduction

Cancer remains one of the leading causes of mortality worldwide, with a steadily increasing global burden driven by demographic changes and continued exposure to risk factors. Epidemiological data indicate that both the incidence of new cancer cases and cancer-related mortality remain at very high levels, and projections for the coming decades predict a further substantial increase in the global cancer burden [1,2,3,4]. Therefore, the identification of novel anticancer agents exhibiting high tumor selectivity and reduced systemic toxicity remains a major focus of contemporary research. In this context, the search for new natural and synthetic compounds with potential anticancer activities represents a central objective for many research groups.

Among the diverse classes of bioactive substances studied to date, compounds containing a lactone ring in their molecular framework have emerged as one of the most promising groups because of their broad spectrum of biological activities [5,6,7]. These include antiviral [8,9], antifungal [10,11], antibacterial [12,13], antifeedant [14,15], antithrombotic [16], antiglycation [17], antioxidant [18,19], and anti-inflammatory effects [20].

In view of the growing global cancer burden and the urgent need for more effective and selective therapies, both natural and synthetic lactones, particularly those bearing an aromatic ring, are increasingly being recognized as valuable lead compounds for the development of novel antitumor agents [21,22,23].

Several β-aryl-substituted γ- and δ-lactones have been reported to exhibit antiproliferative activities against human and canine cell lines [24,25,26]. Aromatic aldehydes are widely used as starting materials for the synthesis of these compounds because many of them are inexpensive and readily available industrial commodities [27,28,29].

The biological activity of these compounds is often strongly influenced by their stereochemistry. The presence of chiral centers may significantly affect interactions with biological targets, leading to differences in potency, selectivity, pharmacokinetic properties, and toxicity among individual stereoisomers [30,31]. In many cases, enantiomers of the same compound exhibit distinct biological profiles, with one stereoisomer displaying superior therapeutic activity compared with its counterpart. In the case of antiproliferative lactones, the importance of stereochemistry has been demonstrated for the enantiomers of goniothalamine, where the (*S*)-enantiomer exhibited approximately 1600 times greater antiproliferative activity than (*R*)-goniothalamine against the 786-0 renal cancer cell line [32].

In our previous study, we reported the synthesis of a series of novel vanillin-derived halolactones in racemic form and evaluated their antiproliferative, hemolytic, antioxidant, and anti-inflammatory activities [33]. We also investigated the interactions of the most active compound with cell membranes using model lipid systems and ATR-FTIR spectroscopy [34]. These studies identified the *cis*- and *trans*-δ-iodo-γ-lactones as the most biologically active derivatives among the synthesized vanillin-derived halolactones, highlighting their potential as promising anticancer candidates.

To determine the influence of stereochemistry on the biological activity of these compounds, in the present work, we prepared enantiomeric pairs of *cis*- and *trans*-4-(4′-hydroxy-3′-methoxyphenyl)-5-(1-iodoethyl)dihydrofuran-2-one using a chemoenzymatic synthesis based on a *Candida antarctica* lipase B (CALB)-catalyzed transesterification step. The isolated enantiomers were subsequently evaluated for their effects on cell viability in a panel of human cancer cell lines. To further characterize the cellular effects of the tested compounds, DNA fragmentation was assessed using the TUNEL (terminal deoxynucleotidyl transferase dUTP nick-end labeling) assay, followed by a flow cytometric analysis of cell cycle distribution.

## 2. Results and Discussion

### 2.1. Enzymatic Kinetic Resolution of Racemic Allylic Alcohol ***1***

Our strategy for obtaining the enantiomers of β-aryl-δ-iodo-γ-lactones **7a** and 7**b** was based on the kinetic resolution of their precursor, racemic (*E*)-4-(4′-benzyloxy-3′-methoxyphenyl)but-3-en-2-ol (**1**). This compound was synthesized from vanillin according to a previously reported three-step procedure [33]. In our effort to obtain both enantiomers of alcohol **1**, we referred to previous studies on the lipase-catalyzed kinetic resolution of its analogues derived from simple aromatic aldehydes, such as benzaldehyde, *p*-methylbenzaldehyde, cuminaldehyde, 2,5-dimethylbenzaldehyde, and piperonal [24,35,36].

The first step of the kinetic resolution studies involved selecting the optimal conditions for the enzymatic transesterification of alcohol **1** in terms of both the biocatalyst and the acyl donor. The reactions were performed in diisopropyl ether (DIPE) at room temperature using 25 mg of substrate, with isopropenyl acetate or vinyl propionate as acyl donors. The progress of the reactions and the enantiomeric purity of the unreacted substrate **1** and products **2** and **3** were monitored by chiral high-performance liquid chromatography (HPLC) analysis, with racemic alcohol **1** and racemic acetate **2** and propionate **3** used as the reference compounds. Esters **2** and **3** were synthesized by Steglich esterification of alcohol **1** with acetic and propionic acid, respectively (Section 3.4.2). Three commercially available lipase preparations were evaluated for their catalytic efficiency and enantioselectivity. Lipozyme^®^ (lipase from *Mucor miehei*) and CALB (lipase B from *Candida antarctica*) were applied in immobilized forms, whereas Amano Lipase PS (lipase from *Burkholderia cepacia*) was used as a free enzyme. The results are summarized in Table 1.

The selection of the optimal reaction conditions was guided by three key criteria: high enantiomeric excesses of both the substrate and the product, conversion close to the theoretical optimum for kinetic resolution (approximately 50%), and short reaction times. In the reaction with isopropenyl acetate, CALB proved to be the most effective and enantioselective biocatalyst (E > 100), affording acetate (*R*)-**2** with 93% ee and the unreacted alcohol (*S*)-**1** with 99% ee at 54% conversion after 6 h (entry 5). In contrast, the reactions catalyzed by Amano Lipase PS and Lipozyme^®^ proceeded much more slowly, reaching only 34% and 12% conversion after 24 h, respectively (entries 1 and 3). Consequently, they exhibited lower enantioselectivity (E = 44 and 18 for Amano PS and Lipozyme^®^, respectively) and yielded alcohol (*S*)-**1** with only 43% and 12% ee, respectively.

Improved kinetic resolution parameters for all three tested enzymes were observed when vinyl propionate was used as the acyl donor (entries 2, 4 and 6). In the case of Amano Lipase PS and Lipozyme^®^, a significant increase in enantioselectivity was observed (E > 100). The conversion of alcohol (*S*)-**1** increased to 46% for Amano Lipase PS and 53% for Lipozyme^®^, which led to significantly higher enantiomeric excesses of the unreacted alcohol (71% and 95%, respectively) and the formed propionate (*R*)-**3** (99% for both enzymes) after 24 h of reaction. For CALB, a further increase in the enantiomeric excess of propionate (*R*)-**3** from 93% to 99% was observed, while the alcohol retained 99% ee and the reaction time was significantly shorter (6 h) compared with those of the other lipases tested.

**Scheme 1:**
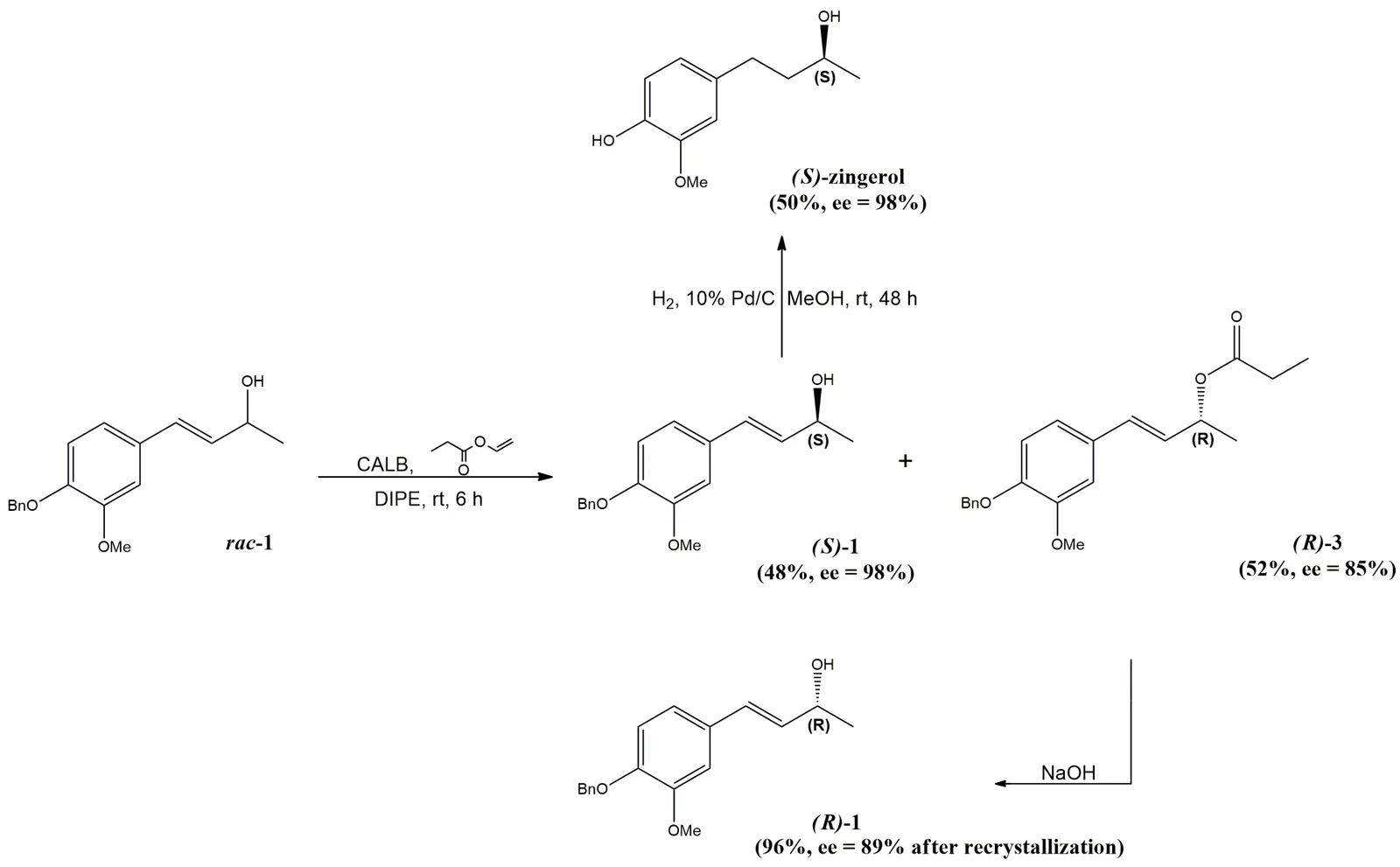
Preparation of enantiomers of allylic alcohol **1** via CALB-catalyzed transesterification and conversion of (*S*)-**1** to (*S*)-zingerol to confirm absolute configuration.

Based on these results, the CALB/vinyl propionate reaction system was selected as optimal for the larger-scale kinetic resolution of racemic alcohol **1**. After 6 h, the products were separated by flash chromatography to afford the unreacted alcohol (*S*)-**1** in 48% yield and 98% ee and propionate (*R*)**-3** in 52% yield and 85% ee (Scheme 1). The purified propionate (*R*)-**3** was subsequently hydrolyzed under alkaline conditions, affording the complementary (*R*)-enantiomer of **1** in high yield (96%) and 89% ee after recrystallization from *n*-hexane. The slightly lower ee of propionate **3** observed during scale-up may result from minor changes in reaction parameters relative to the initial screening experiments, such as mixing efficiency, substrate concentration, or water content, all of which can influence the selectivity of the CALB-catalyzed kinetic resolution. Nevertheless, the reaction maintained high enantioselectivity, enabling the efficient preparation of both enantiomers of alcohol **1** in high optical purity.

The absolute configurations of enantiomeric alcohols (*R*)-**1** and (*S*)-**1** were assigned based on the empirical Kazlauskas’ rule, which predicts the preferential esterification of one enantiomer of a secondary alcohol by lipases [37]. According to this rule, enantioselectivity during lipase-catalyzed kinetic resolution arises primarily from differences in the steric size of the substituents at the stereogenic center. Assuming the priority order OH > large substituent > medium substituent, most lipases, including CALB, preferentially catalyze the transesterification of the (*R*)-enantiomer. This rule was empirically confirmed in our previous study on the kinetic resolution of allylic alcohols of the (*E*)-4-arylbut-3-en-2-ol type, bearing phenyl or *p*-methylphenyl substituents [35], and was extended to analogues with *p*-isopropylphenyl, 2,5-dimethylphenyl, and 1,3-benzodioxole moieties [24,36].

The same reasoning was applied herein to analogous alcohol **1**, derived from vanillin. Considering its close structural similarity to the previously studied compounds, the use of CALB as the biocatalyst under the same transesterification conditions, and the observed signs of specific rotation for the enantiomers of alcohol, we assigned the *S*-configuration to the levorotatory, slower-reacting enantiomer and the *R*-configuration to the dextrorotatory, faster-reacting enantiomer obtained after the alkaline hydrolysis of the corresponding propionate (*R*)-**3**.

To obtain independent experimental confirmation of this assignment, the slower-reacting enantiomer of alcohol **1** was converted to a compound of known absolute configuration, namely zingerol (Scheme 1). Catalytic hydrogenation over Pd/C in methanol was applied to the simultaneous reduction of the double bond and the cleavage of the benzyl protective group. After purification by flash chromatography and NMR analysis, confirming the structure of synthesized zingerol (spectroscopic data provided in Section 3.4.4), its specific rotation was measured in EtOH and compared with the literature values [38]. The positive sign of the specific rotation for the zingerol synthesized herein and for known (*S*)-zingerol, combined with comparable magnitudes (${\left[\alpha \right]}_{D}^{20}$ = +13.2 versus the literature value, ${\left[\alpha \right]}_{D}^{20}$ = +14.1), confirmed the (*S*)-configuration of the slower-reacting alcohol **1**. Consequently, the faster-reacting enantiomer was assigned the (*R*)-configuration. This chemical correlation provides independent support for the stereochemical assignment of enantiomeric alcohols, **1**, and aligns with the preliminary assignment based on Kazlauskas’ rule.

### 2.2. Synthesis of Enantiomerically Enriched Iodolactones ***7a*** and ***7b***

**Scheme 2:**
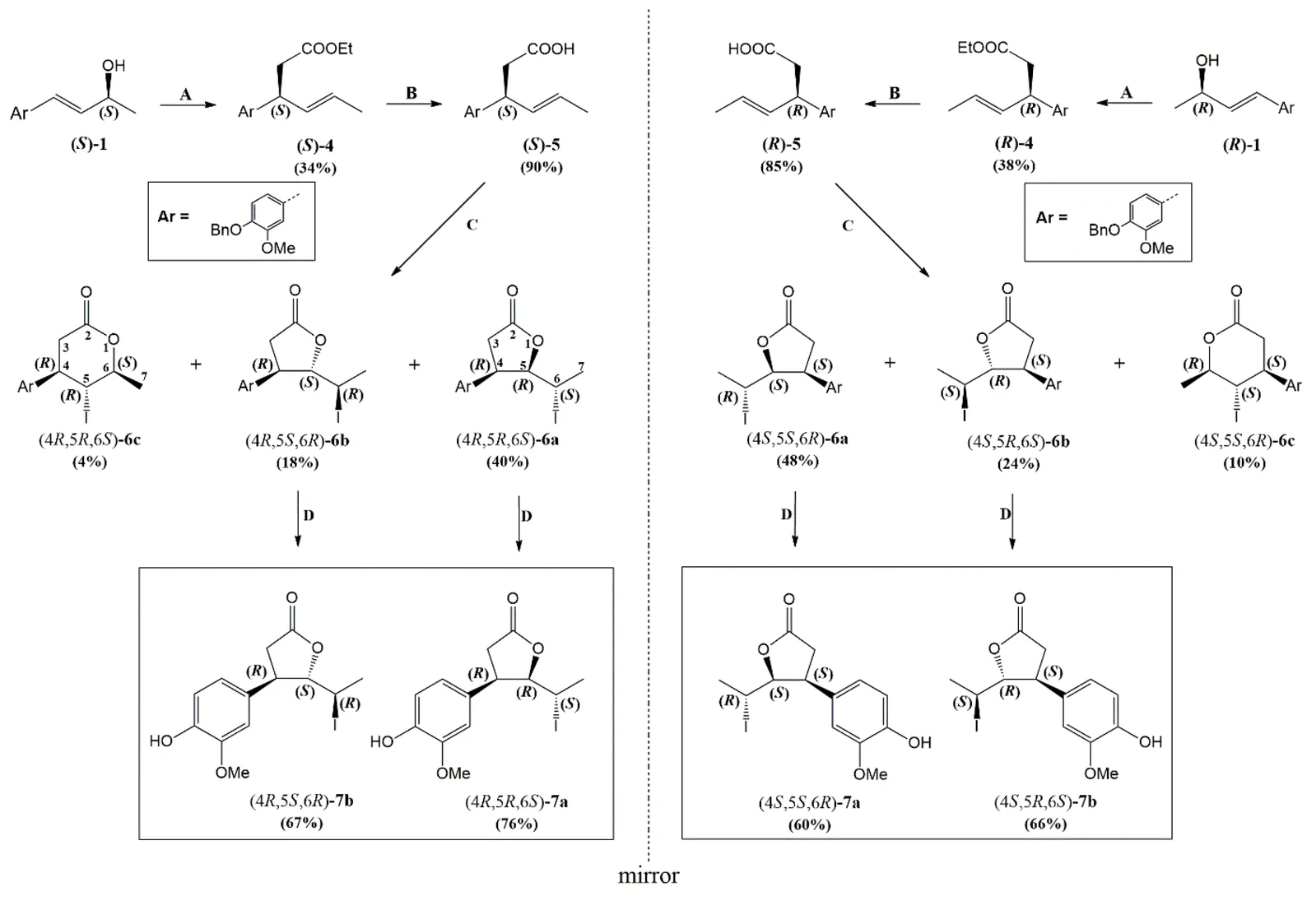
Synthesis of enantiomerically enriched iodolactones, *cis*-(4*R*,5*R*,*6S*)-**7a**, *cis*-(4*S*,5*S*,6*R*)-**7a** and *trans*-(4*R*,5*S*,6*R*)-**7b**, and *trans*-(4*S*,5*R*,6*S*)-**7b,** from the enantiomeric allylic alcohols (*S*)-**1** and (*R*)**-1.** Reagents and conditions: (**A**) MeC(OEt)_3_, EtCOOH, 138 °C, 6 h; (**B**) 10% NaOH, EtOH, reflux, 5 h; (**C**) I_2_/KI, Et_2_O, NaHCO_3_, r.t., 20 h; (**D**) 1 M BCl_3_/CH_2_Cl_2_, −68 °C, 20 min.

Having obtained both (*S*) and (*R*) enantiomers of alcohol **1**, we synthesized the final enantiomerically enriched iodolactones **7a** and **7b** via a four-step pathway previously described for racemic lactones [33]. The sequence comprised a Claisen–Johnson rearrangement, followed by hydrolysis, and iodolactonization of acids (*S*)-**5** and (*R*)-**5**, followed by deprotection (Scheme 2).

From a stereochemical perspective, the key step of this synthetic pathway was the formation of γ,δ-unsaturated esters (*S*)-**4** and (*R*)-**4** during the Claisen–Johnson rearrangement. In this process, the allylic alcohol reacts with triethyl orthoacetate to generate an allyl vinyl ether, which undergoes a [3,3]-sigmatropic rearrangement upon heating at 138 °C to afford the γ,δ-unsaturated ester. Mechanistic studies on this reaction [39] demonstrated that chirality transfer from C-2 of the allylic alcohol to the γ,δ-unsaturated ester proceeds with high stereoselectivity. For (*E*)-configured starting alcohols, both the retention of the double-bond geometry (as confirmed by coupling constants between olefinic protons (*J* = 15.3 Hz) in the ^1^H NMR spectra) and the retention of the absolute configuration at the newly formed stereogenic center located at the benzylic C-3 position are observed. These stereochemical findings were previously reported for analogous β-aryl-γ,δ-unsaturated esters bearing different substitution patterns on the aromatic ring formed via the Claisen–Johnson rearrangement [24,35,36].

According to this mechanism, in the present study, the alcohol (*S*)-**1** (ee = 98%) was converted into the γ,δ-unsaturated ester (*S*)-**4** (ee = 95%), whereas the alcohol (*R*)-**1** (ee = 89%) afforded the ester (*R*)-**4** (ee = 76%). The enantiomeric (*S*)- and (*R*)-esters **4** were further converted to iodolactones **6a**–**c** via hydrolysis and subsequent iodolactonization. Similar to the synthesis of racemic iodolactones reported previously [33], the reaction afforded mixtures of *cis*-δ-iodo-γ-lactone (40–48%), *trans*-δ-iodo-γ-lactone (18–24%) and minor amounts of γ-iodo-δ-lactone (4–10%), which were successfully isolated and purified by flash chromatography (Scheme 2). Their structures were confirmed by comparison of their spectroscopic data with those of their racemic counterparts obtained in our previous study [33]. The configurations of the stereogenic centers were established based on the iodolactonization mechanism, which has been thoroughly described for analogous iodolactones obtained through a similar synthetic procedure [24]. The configuration (*R*) or (*S*) at C-4 in both γ-lactone isomers is determined by the configuration of the starting acid; the apparent change in configuration relative to the precursor is not due to inversion but reflects the different priority of substituents. The reaction mechanism involves electrophilic attack by iodine on the double bond, leading to the formation of an iodonium ion. Subsequently, the carboxylate ion undergoes nucleophilic attack at C-5 from the side opposite the iodonium ion, resulting in an antiperiplanar arrangement of the C–O and C–I bonds. For the *cis*-γ-lactone formed from the (*S*)-acid, this results in the *R*-configuration at C-5 and the *S*-configuration at C-6. By analogous reasoning, the *trans* isomer is assigned the 5*S*,6*R* configurations. Lactones obtained from the (*R*)-acids possess the opposite configurations at the corresponding stereogenic centers (Scheme 2).

The final step of the synthesis was the selective removal of the benzyl protecting group to afford enantiomeric pairs of the target *cis*-(**7a**) and *trans*-δ-iodo-γ-lactones (**7b**). In our previous studies, selective deprotection of the benzyl group proved challenging: several commonly used debenzylation methods, including catalytic hydrogenolysis over Pd/C, reaction with 33% HBr in acetic acid, treatment with concentrated HCl under reflux, and the use of aqueous NaBrO_3_ and Na_2_S_2_O_3_ solutions, resulted either in the formation of inseparable mixtures or in the partial isomerization of the *cis*-δ-iodo-γ-lactone to the corresponding *trans*-isomer. Ultimately, FeCl_3_-mediated debenzylation in dry dichloromethane afforded the desired phenolic lactones, but the isolated yields were low (16–28%), even after the optimization of the work-up procedure. To improve the overall yield of the optically active products, in the present work, we employed a BCl_3_-mediated protocol applied by Bouton et al. for the synthesis of azanucleoside derivatives [40]. Treatment of the enantiomeric benzyl-protected iodolactones **6a** and **6b** with a 1 M solution of BCl_3_ in dry dichloromethane at −68 °C afforded clean and selective debenzylation after 20 min (Scheme 2). After work-up, purification by flash chromatography and subsequent recrystallization from *n*-hexane, pairs of deprotected enantiomerically enriched *cis*- and *trans*-δ-iodo-γ-lactones (**7a** and **7b**) with ee = 64–87% were obtained in significantly improved yields (60–76%) compared to the previously applied FeCl_3_ protocol.

### 2.3. Effects of Enantiomerically Enriched Iodolactones ***7a*** and ***7b*** on Cell Viability

The effects of enantiomeric pairs of *cis*- and *trans*-δ-iodo-γ-lactones **7a** and **7b** on cell viability were evaluated in vitro using an MTT assay against human cancer cell lines, including melanoma (LM-MEL-75), gastric adenocarcinoma (EPG85-257RDB), and ovarian carcinoma (A2780), as well as normal human dermal fibroblasts (NHDFs) as a non-cancerous reference cell line. Racemic mixtures of the target lactones (*rac*-**7a** and *rac*-**7b**) were included as reference standards, given their previously established bioactivity against the evaluated cell lines [34]. Doxorubicin was used as a positive control. The results are expressed as IC_50_ values and summarized in Table 2.

In all tests against cancer cells, except for some comparisons in the A2780 cell line, the enantiomers of *trans*-δ-iodo-γ-lactone **7b** exhibited significantly stronger cell-viability-reducing effects than the corresponding enantiomers of *cis*-δ-iodo-γ-lactone **7a**. For *cis*-oriented stereoisomers, the IC_50_ values remained within the range of approximately 18–36 μM, whereas *trans* stereoisomers exhibited pronounced cell-viability-reducing activity in the concentration range of 4–14 μM. These determined IC_50_ values were not statistically different from those determined for doxorubicin, used as the reference drug. The highest activity was observed for *trans* enantiomers (4*R*,5*S*,6*R*)-**7b** and (4*S*,5*R*,6*S*)-**7b** against the EPG85-257RDB multidrug-resistant gastric adenocarcinoma cell line, with IC_50_ values of 3.98 ± 2.60 μM and 5.08 ± 1.30 μM, respectively. These findings demonstrate that both compounds reduce the viability of EPG85-257RDB cells. However, because a matched drug-sensitive parental cell line was not included in the present study, a resistance index could not be calculated, and these results do not establish whether the MDR phenotype affects the sensitivity of these cells to the investigated compounds.

Importantly, in contrast to doxorubicin, all of the investigated iodolactones exhibited lower cell-viability-reducing activity against normal NHDFs than against the tested cancer cells, indicating in vitro selectivity under the experimental conditions used. This selectivity was evaluated using the selectivity index (SI) (Table 3).

For the enantiomers of *cis*-δ-iodo-γ-lactone **7a**, SI values ranged from 1.52 to 2.27, indicating only modest selectivity. In contrast, both enantiomers of *trans*-δ-iodo-γ-lactone **7b** demonstrated improved selectivity profiles, particularly for the EPG85-257RDB cell line (SI = 10.19 for (4*S*,5*R*,6*S*)-**7b** and 9.22 for (4*R*,5*S*,6*R*)-**7b**). However, these SI values represent an initial in vitro comparison based on a single non-malignant cell model and cannot be interpreted as evidence of systemic safety. Further evaluation using additional tissue-relevant non-malignant cell models and longer exposure times is required.

Taken together, the present findings demonstrate that the key structural feature governing the cell-viability-reducing effects of vanillin-derived iodolactones is the relative orientation of substituents on the γ-lactone ring. The *trans* configuration appears to be favorable, as it combines enhanced cell-viability-reducing activity with improved selectivity against cancer cells. A similar stereochemical preference has been previously reported by Gładkowski et al. for chiral δ-iodo-γ-lactones derived from simple aldehydes, including cuminaldehyde, 2,5-dimethylbenzaldehyde, and piperonal, in which the *trans* stereoisomers consistently demonstrated higher in vitro antiproliferative activity than the corresponding *cis* stereoisomers against the Jurkat, D17, CL-1, and CLBL1 cell lines [24].

The higher activity of the *trans* isomers compared to their *cis* counterparts observed in the present study may be related to differences in their three-dimensional arrangement and conformational preferences, which can influence their interactions with biological membranes and the accessibility of potential cellular targets. In our previous studies, racemic **7b** was found to incorporate into biological membranes, concentrating mainly in the hydrophilic part of the bilayer [34]. The same observations were made for the chiral *trans* δ-bromo-γ-lactones derived from 2,5-dimethylbenzaldehyde, which also proved to be the most active isomers among this group of bromolactones [25]. These observations suggest that perturbation of membrane organization may represent one of the factors contributing to the cell-viability-reducing activity of *trans* isomers. Moreover, the *trans* configuration positions substituents at the lactone ring, such as aryl and haloalkyl chains, in an orientation that may fit target protein binding pockets better than the *cis* form. For example, both enantiomers of piperonal-derived *trans* β-aryl-δ-iodo-γ-lactones exhibited strong hydrophobic interactions with HSA, which correlated with their high antiproliferative and proapoptotic activities—an effect not observed for *cis* isomers [41].

When evaluating the impact of absolute configuration on the activity of the vanillin-derived lactones, we found that, even though the samples differed in enantiomeric excess, both were significantly enriched in one enantiomer. Specifically, a 64% ee corresponded to an 82:18 enantiomeric ratio, whereas the other sample exhibited an enantiomeric composition of 98.5:1.5 (ee = 97%). Despite these markedly different enantiomeric compositions, both samples displayed similar activity; under the experimental conditions, no statistically significant differences were observed between the corresponding enantiomerically enriched forms. The influence of stereochemistry on activity has been previously observed for other aryl aldehyde-derived β-aryl-δ-iodo-γ-lactones. An evaluation of an enantiomeric pair of *trans*-β-aryl-δ-iodo-γ-lactone derived from piperonal revealed that the (4*S*,5*R*,6*S*)-enantiomer displayed notably higher binding affinity to biological macromolecules (such as human serum albumin) and higher cytotoxicity against a set of canine lymphoma and/or leukemia cell lines than its counterpart [24,41]. Similarly, among cuminaldehyde-derived δ-iodo-γ-lactones stereoisomers, those bearing the (4*S*) configuration were more active against selected canine cancer cell lines than those possessing the (4*R*) configuration [42]. In contrast, for the *trans* β-aryl-δ-iodo-γ-lactone derived from 2,5-dimethylbenzaldehyde, the (4*R*,5*S*,6*R*)-enantiomer exhibited clearly more potent cytotoxic effects [43].

Considering the structure–activity relationships within the group of β-aryl-δ-iodo-γ-lactones synthesized by our research group thus far [24,33,41,42,43], the *trans* configuration appears to be a key feature influencing activity. Additionally, the presence of oxygen-containing functional groups on the benzene ring—such as the dioxole ring in piperonal-derived lactones and the hydroxy and methoxy substituents in vanillin-derived lactones—is favorable. Generally, iodolactones with bulkier aryl substituents (such as 4-isopropylphenyl, 2,5-dimethylphenyl, or 3-methoxy-4-hydroxyphenyl rings) exhibit higher activity than those with unsubstituted or 4-methyl-substituted phenyl rings.

### 2.4. Assessment of DNA Fragmentation by the TUNEL Assay

While the ability of lactone derivatives to reduce cancer cell viability provides an initial indication of their biological activity, elucidating the cellular mechanisms underlying these effects is essential for evaluating their suitability as anticancer agents. Such mechanistic insight enables the identification of key cellular targets and structure–activity relationships governing biological responses [44,45].

To further characterize the cellular effects associated with the reduction in cell viability induced by the vanillin-derived iodolactones, the compound–cell line combination for subsequent analyses was selected based on the results of the MTT assay. The (4*R*,5*S*,6*R*)-**7b** enantiomer exhibited the strongest cell-viability-reducing activity against the EPG85-257RDB cell line together with a high selectivity index. Notably, EPG85-257RDB is a multidrug-resistant gastric adenocarcinoma cell line, making the pronounced activity of (4*R*,5*S*,6*R*)-**7b** against this model particularly relevant. Therefore, this compound was selected as the most promising candidate for preliminary characterization of its cellular effects, while EPG85-257RDB was selected as the most sensitive cancer cell model.

One of the mechanisms of action of anticancer compounds is the induction of programmed cell death, known as apoptosis, which can proceed through multiple distinct molecular pathways. However, a common downstream hallmark of several apoptotic pathways is internucleosomal DNA fragmentation, resulting in DNA strand breaks with exposed 3′-hydroxyl termini. This endpoint can be effectively detected using the TUNEL assay, which enables the identification of DNA strand breaks by enzymatic labeling of free 3′-OH termini with terminal deoxynucleotidyl transferase (TdT) and labeled dUTP nucleotides. The resulting signal enables the detection of cells exhibiting DNA strand breaks. However, TUNEL positivity is not specific for apoptosis, as DNA fragmentation may also occur during other forms or late stages of cell death [46]. Therefore, in the present study, the proportion of TUNEL-positive cells was interpreted as an indicator of DNA fragmentation rather than as definitive evidence of apoptosis.

The results demonstrated a concentration-dependent increase in TUNEL-positive EPG85-257RDB cells following treatment with (4*R*,5*S*,6*R*)-**7b** (Table 4). The proportion of TUNEL-positive cells increased progressively with increasing concentrations of the tested compound, indicating enhanced DNA fragmentation. The strongest effect was observed at 20 µg/mL, where 74.10 ± 4.26% of cells were TUNEL-positive, indicating DNA fragmentation in nearly three-quarters of the analyzed cell population. Considering the IC_50_ value of 3.98 ± 2.60 µM determined for (4*R*,5*S*,6*R*)-**7b** in EPG85-257RDB cells, the concentrations of 1 and 2 µg/mL (2.76 and 5.52 µM) correspond to approximately 0.69× and 1.39× IC_50_, respectively. These concentrations are therefore the most relevant for interpretations of the cellular effects associated with reduced cell viability. In contrast, the extensive DNA fragmentation observed at the highest concentrations may partly represent the secondary consequences of severe cytotoxicity.

EPG85-257RDB cells are resistant to commonly used chemotherapeutic agents and are widely used as a model of MDR cancer cells [47,48]. The observed increase in TUNEL-positive cells indicates that treatment with the compound is associated with increased DNA fragmentation in EPG85-257RDB cells. These findings suggest that DNA fragmentation may contribute to the reduced cell viability observed in the MTT assay.

### 2.5. Analysis of Cell Cycle Distribution

**Figure 1:**
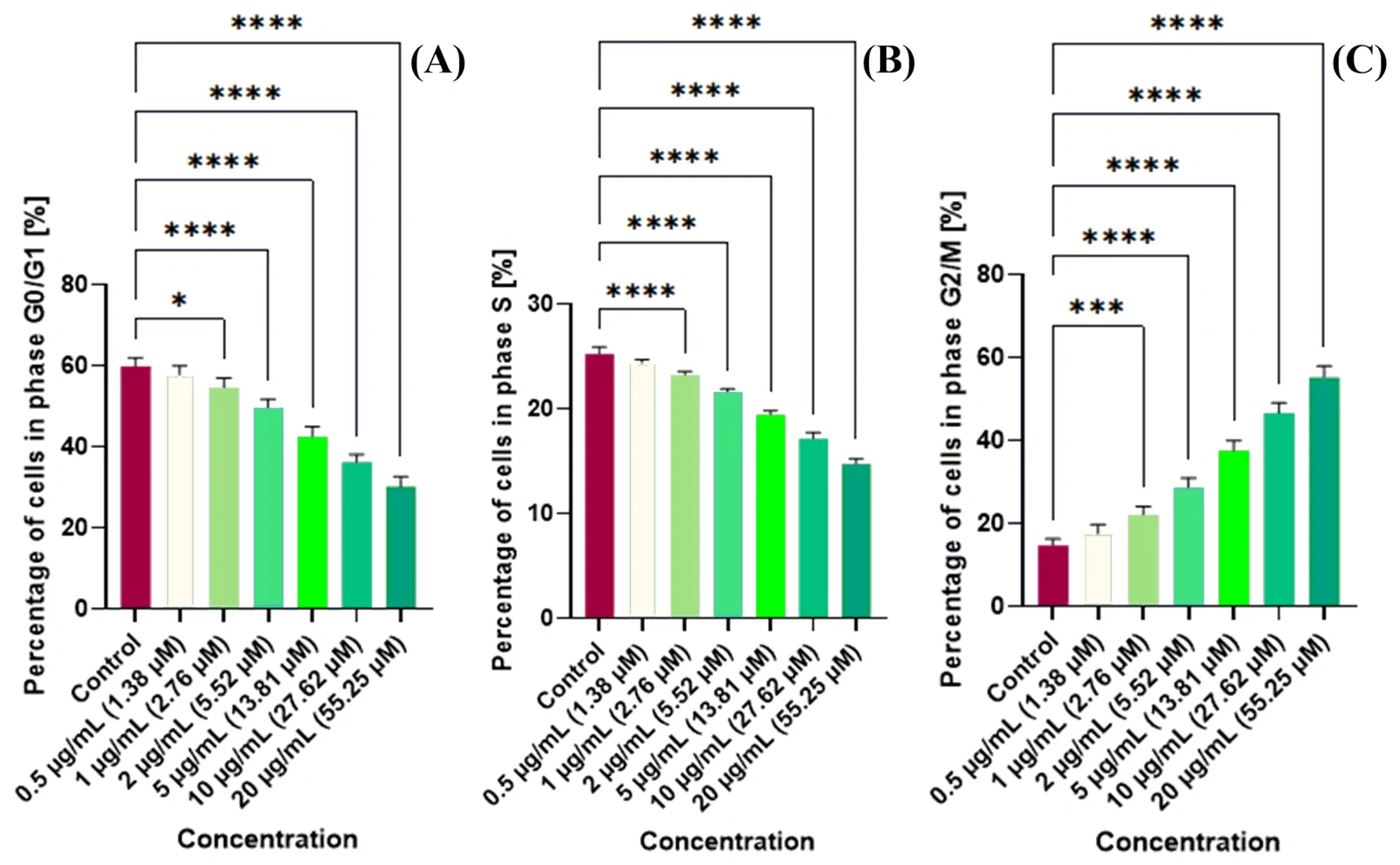
Distribution of cells across the cell cycle phases in EPG85-257RDB cells after 24 h of treatment with compound (4*R*,5*S*,6*R*)-**7b** (0.5–20 µg/mL, corresponding to 1.38–55.25 µM): G0/G1 (**A**), S (**B**) and G2/M (**C**). Data represent mean values ± SD from four independent biological replicates (n = 4); no technical replicates were performed. Statistical analysis was performed using ANOVA followed by Dunnett’s multiple comparison test for comparisons with the control group. Statistical significance relative to the control group was denoted as follows: * *p* < 0.05, *** *p* < 0.001 and **** *p* < 0.0001.

The effect of the (4*R*,5*S*,6*R*)-enantiomer of iodolactone **7b** on cell cycle distribution in EPG85-257RDB cells was evaluated by a flow cytometric analysis of DNA content following propidium iodide staining (Figure 1). Flow cytometry enables the quantitative assessment of cellular DNA content and discrimination of cell populations in the G0/G1, S, and G2/M phases, enabling evaluation of treatment-associated changes in cell cycle distribution. Representative DNA content histograms from the flow cytometric analysis are provided in the Supplementary Materials (Figure S28).

The analysis of cell cycle distribution revealed a significant, concentration-dependent effect of the tested iodolactone on EPG85-257RDB cells. In control cells, the majority of the population (59.93 ± 2.11%) was in the G0/G1 phase with 25.25 ± 0.66% in the S phase and 14.83 ± 1.48% in the G2/M phase. Exposure to increasing concentrations of the compound resulted in a progressive redistribution of the cell population, characterized by a continuous decrease in the proportion of cells in the G0/G1 phase and a concomitant accumulation of cells in the G2/M phase.

Even moderate concentrations (1–5 µg/mL) induced significant cell cycle redistribution, with progressively stronger effects at higher concentrations. Specifically, the proportion of cells in the G0/G1 phase decreased in a concentration-dependent manner from 59.93 ± 2.11% in control cells to 30.30 ± 2.43% following treatment with 20 µg/mL. Similarly, a significant reduction in the S phase fraction was observed, from 25.25 ± 0.66% (control) to 14.75 ± 0.50% (20 µg/mL), indicating impaired DNA synthesis progression. The most pronounced effect was observed in the G2/M phase, where the proportion of cells increased from 14.83 ± 1.48% in control cells to 55.25 ± 2.74% after treatment with the compound at 20 µg/mL. The cell cycle effects observed at concentrations close to the IC_50_ value are considered more relevant to the primary cellular response, whereas the pronounced changes observed at 10–20 µg/mL (27.62–55.25 µM) should be interpreted cautiously, as they may partly reflect secondary effects associated with extensive cytotoxicity.

The observed cell cycle perturbations were accompanied by a concentration-dependent increase in TUNEL-positive cells, indicating enhanced DNA fragmentation. G2/M accumulation may reflect a cellular response to the tested compound and be associated with impaired cell cycle progression. However, these parallel concentration-dependent changes do not establish a causal relationship between G2/M accumulation and DNA fragmentation. Therefore, these findings are interpreted as concurrent cellular responses to treatment rather than evidence that G2/M accumulation directly induces cell death, while the observed DNA fragmentation may contribute to the reduction in cell viability detected in the MTT assay.

The pronounced effect observed in EPG85-257RDB cells, in a model of MDR gastric cancer, supports further investigation of the (4*R*,5*S*,6*R*)-enantiomer of **7b** in drug-resistant cancer models. Comparative studies using matched drug-sensitive and -resistant cell lines will be required to determine whether the MDR phenotype influences the activity of this compound.

To date, several aromatic aldehyde-derived halolactones have been investigated in greater detail, revealing their ability to interfere with the key processes regulating cancer cell survival and death. The antiproliferative activity of δ-iodo-γ-lactones derived from cuminaldehyde was associated with the induction of programmed cell death through activation of the intrinsic apoptotic pathway accompanied by caspase activation [42]. Similarly, enantiomeric *trans*-β-aryl-δ-iodo-γ-lactones obtained from 2,5-dimethylbenzaldehyde were found to promote apoptosis in cancer cells through the modulation of apoptosis-related proteins, including the downregulation of Bcl-2 and Bcl-xL. Moreover, in canine cancer cell lines, these compounds were shown to engage additional apoptotic signaling pathways, further enhancing their proapoptotic activity [43].

## 3. Materials and Methods

### 3.1. Chemicals and Enzymes for Synthesis

Vinyl propionate (98%), isopropenyl acetate (99%), triethyl orthoacetate (97%), *N*,*N*′-dicyclohexylcarbodiimide (DCC, 99%), 4-(dimethylamino)pyridine (DMAP, ≥99%), palladium on carbon (Pd/C), boron trichloride solution (1 M in dry dichloromethane), isopropanol (UHPLC, ≥99%), *n*-hexane (UHPLC, ≥99%), methanol (UHPLC, ≥99%), diisopropyl ether (DIPE, ≥99%), chloroform (ethanol-free, ≥99.5%), anhydrous dichloromethane (99.8%) DMSO (biological grade), Amano Lipase PS from *Burkholderia cepacia* (≥30,000 U/mg), Lipozyme^®^ immobilized lipase from *Mucor miehei* (30 U/g) and Lipase B from *Candida antarctica*, immobilized on acrylic resin (CALB, >5000 U/g), were obtained from Sigma-Aldrich^®^ (Steinheim or Darmstadt, Germany).

### 3.2. Chemicals for Biological Studies

Propidium iodide (PI, ≥94%) and ribonuclease A (RNase A) were purchased from MedChemExpress (Monmouth Junction, NJ, USA). Paraformaldehyde (≥95%) and hydrogen peroxide (H_2_O_2_, analytical grade) were purchased from POCH (Gliwice, Poland). Xylene (>98%) was purchased from Sanlab (Warsaw, Poland).

Roswell Park Memorial Institute cells (RPMI-1640), L-glutamine, streptomycin, penicillin, Fetal Bovine Serum (FBS), trypsin, ethylenediaminetetraacetic acid (EDTA), doxorubicin and 3-(4,5 dimethylthiazol-2-yl)-2,5-diphenyltetrazolium bromide (MTT) were purchased from Sigma-Aldrich^®^ (Steinheim, Germany). Dulbecco′s Modified Eagle′s Medium (DMEM) was obtained from Thermo Fisher Scientific (Waltham, MA, USA).

Phosphate-buffered saline (PBS), trypsin, trypsin–EDTA solution (0.25%) and hematoxylin were purchased from Sigma-Aldrich (St. Louis, MO, USA).

### 3.3. Analysis and Purification of the Synthesized Compounds

The reaction progress was followed by thin-layer chromatography (TLC) carried out on 0.2 mm aluminum plates coated with silica gel 60 F254 (Merck, Darmstadt, Germany). The chromatograms were visualized by spraying the plates with a 1% solution of Ce(SO_4_)_2_ and 2% H_3_[P(Mo_3_O_10_)_4_] in 10% H_2_SO_4_, followed by heating the plates at 120–200 °C.

Nuclear magnetic resonance (NMR) spectra, including ^1^H NMR, ^13^C NMR, ^13^C DEPT-135, COSY, HMQC and HMBC spectra (Supplementary Materials; Figures S1–S14), were recorded on either a JEOL 400 MHz spectrometer (JEOL Ltd., Tokyo, Japan) or a Bruker Avance III HD 600 MHz spectrometer (Bruker BioSpin GmbH, Rheinstetten, Germany). Samples were dissolved in CDCl_3_ and chemical shifts were referenced to the residual solvent signal (δ_H_ = 7.26, δ_C_ = 77.00 ppm).

Infrared (IR) spectra (Supplementary Materials; Figures S15 and S16) were acquired using a Nicolet iS10 FTIR Spectrometer (Thermo Fisher Scientific™, Waltham, MA, USA), equipped with a monolithic diamond ATR crystal attachment.

High-resolution mass spectra (HRMS) (Supplementary Materials; Figures S17 and S18) were recorded on a Bruker Daltonics ESI-Q-TOF maXis impact mass spectrometer (Bruker, Billerica, MA, USA) using positive electrospray ionization (ESI) mode.

Specific optical rotations were measured for samples dissolved in dichloromethane (concentration denoted in g/100 mL) on a Jasco P-2000-Na digital polarimeter with an intelligent Remote Module (iRM) controller (JASCO Corporation, Tokyo, Japan).

HPLC was performed on a Waters 2695 Alliance instrument with a photodiode array detector, Waters 2996 (Waters Corporation, Milford, MA, USA), using the analytical HPLC chiral column CHIRALPAK AD-H (250 × 4.6 mm, 5 µm) (Daicel Chemical Industries, Osaka/Tokyo, Japan). Separation conditions: mobile phase—*n*-hexane (A) and isopropanol (B); for the enantiomers of compounds **1**–**5**, **6a**,**b** and **7a**,**b**, a flow rate of 1 mL/min was used, and isocratic elution was conducted with A/B = 90:10 (*v*/*v*) for 60 min; for the enantiomers of iodolactone **7c**, a flow rate of 0.5 mL/min was used, and isocratic elution was conducted with A/B = 90:10 (*v*/*v*) for 110 min. Recorded HPLC chromatograms are available in the Supplementary Materials (Figures S19–S27).

Flash chromatography was conducted using the puriFlash^®^ SX520 Plus system (Interchim, Montluçon, France), which includes a gradient pump, a UV detector, and a fraction collector. Samples were dry loaded on a pre-column (puriFlash^®^) and the compounds were separated on puriFlash^®^ SIHP F0012 or F0040 30 µm columns by gradient elution with *n*-hexane/ethyl acetate mixtures (flow rate: 26 mL/min; pressure: 15 mbar).

A summary of the stereochemical assignments, enantiomeric excesses, specific rotations, chiral HPLC data, and the basis for the configuration assignments of the enantiomerically enriched compounds, **1**–**7**, is provided in the Supplementary Materials (Table S1).

### 3.4. Kinetic Resolution of Alcohol 1 by Enzymatic Transesterification

#### 3.4.1. Selection of the Biocatalyst and Acyl Donor

Racemic alcohol **1** (25 mg, 0.09 mmol), lipase (12.5 mg), vinyl propionate or isopropenyl acetate (3 mL) and DIPE (0.5 mL) were placed in a 7 mL vial. The reaction mixture was stirred at room temperature. At selected time intervals (1, 2, 4, 6, 8, and 24 h), 0.3 mL aliquots were withdrawn from the reaction mixture and filtered through PTFE syringe filters (0.22 µm, CHEMLAND, Starogard, Poland). The solvent was evaporated and the residue was dissolved in 1 mL of isopropanol, transferred quantitatively to vials and analyzed by chiral-phase HPLC.

#### 3.4.2. Preparation of Racemic Esters **2** and **3**

Racemic alcohol **1** (0.12 g, 0.42 mmol), DCC (0.12 g, 0.63 mmol) and DMAP (0.082 g, 0.67 mmol) were placed in a round-bottomed flask and 25 mL of ethanol-free chloroform was added. To the stirred mixture, acetic acid (35.40 µL, 0.62 mmol) or propionic acid (46.40 µL, 0.62 mmol) were added dropwise. After 24 h (monitored by TLC), the reaction mixture was filtered, acidified with 0.5 M HCl and extracted with chloroform (3 × 40 mL). The combined organic layers were washed with brine until neutral, dried over anhydrous MgSO_4_ and the solvent was removed under reduced pressure on a rotary evaporator. The crude product was purified by flash chromatography using a gradient elution from 100% *n*-hexane to a *n*-hexane/ethyl acetate (16:1, *v*/*v* for acetate **2** and 19:1, *v*/*v* for propionate **3**). Physical and spectroscopic data for the synthesized esters are provided below:

*(E)-4-(4′-Benzyloxy-3′-methoxyphenyl)but-3-en-2-yl acetate (***2***)*

Yield, 0.06 g (44%); white crystals; mp 76–79 °C; R_f_ = 0.52 (*n*-hexane/acetone 3:1, *v*/*v*); ^1^H NMR (400 MHz, CDCl_3_): δ 1.40 (d, *J* = 6.5 Hz, 3H, C**H_3_**-1), 2.06 (s, 3H, -C(O)C**H_3_**), 3.91 (s, 3H, -OC**H_3_**), 5.15 (s, 2H, -OC**H_2_**Ph), 5.50 (m, 1H, H-2), 6.05 (dd, *J* = 15.9 and 6.9 Hz, 1H, H-3), 6.53 (d, *J* = 15.9 Hz, 1H, H-4), 6.81 (d, *J* = 8.3 Hz, 1H, H-5′), 6.85 (dd, *J* = 8.3 Hz and 1.7 Hz, 1H, H-6′), 6.95 (d, *J* = 1.7 Hz, 1H, H-2′), 7.29 (m, 1H, H-4″), 7.33–7.39 (m, 2H, H-3″, H-5″), 7.40–7.45 (m, 2H, H-2″, H-6″); ^13^C NMR (100 MHz, CDCl_3_): δ 20.41 (C-1), 21.42 (-C(O)**C**H_3_), 55.93 (-O**C**H_3_), 70.94 (-O**C**H_2_Ph), 71.15 (C-2), 109.42 (C-2′), 113.82 (C-5′), 119.72 (C-6′), 126.95 (C-3), 127.21 (C-2″, C-6″), 127.83 (C-4″), 128.52 (C-3″, C-5″), 129.82 (C-1′), 131.46 (C-4), 136.96 (C-1″), 148.13 (C-4′), 149.65 (C-3′), 170.37 (-**C**(O)CH_3_); HRMS (ESI): *m*/*z* calcd for C_20_H_22_O_4_ [M+Na]^+^: 349.1410; found: 349.1421; IR (ATR): ν_max_ = 1727, 1508, 1221, 1139, 1006, 946, 857, 748, 697 cm^−1^.

*(E)-4-(4′-Benzyloxy-3′-methoxyphenyl)but-3-en-2-yl propionate (* **3** *)*

Yield, 0.07 g (47%); white crystals; mp 32–35 °C; R_f_ = 0.46 (*n*-hexane/acetone 3:1, *v*/*v*); ^1^H NMR (400 MHz, CDCl_3_): δ 1.15 (t, *J* = 7.6 Hz, 3H, -C(O)CH_2_C**H_3_**)_,_ 1.40 (d, *J =* 6.5 Hz, 3H, C**H_3_-**1), 2.34 (q, *J* = 7.6 Hz, 2H, -C(O)C**H_2_**CH_3_), 3.91 (s, 3H, -OC**H_3_**), 5.15 (s, 2H, -OC**H_2_**Ph), 5.52 (m, 1H, H-2), 6.05 (dd, *J* = 15.9 Hz and 6.9 Hz, 1H, H-3), 6.52 (d, *J =* 15.9 Hz, 1H, H-4), 6.81 (d, *J* = 8.3 Hz, 1H, H-5′), 6.85 (dd, *J* = 8.3 Hz and 1.8 Hz, 1H, H-6′), 6.95 (d, *J* = 1.8 Hz, 1H, H-2′), 7.29 (m, 1H, H-4″), 7.33–7.39 (m, 2H, H-3″, H-5″), 7.40–7.45 (m, 2H, H-2″, H-6″); ^13^C NMR (100 MHz, CDCl_3_): δ 9.10 (-C(O)CH_2_**C**H_3_), 20.44 (C-1), 27.91 (-C(O)**C**H_2_CH_3_), 55.95 (-O**C**H_3_), 70.92 (C-2), 70.95 (-O**C**H_2_Ph), 109.44 (C-2′), 113.84 (C-5′), 119.70 (C-6′), 127.11 (C-3), 127.21 (C-2″, C-6″), 127.83 (C-4″), 128.53 (C-3″, C-5″), 129.89 (C-1′), 131.37 (C-4), 136.98 (C-1″), 148.12 (C-4′), 149.66 (C-3′), 173.78 (-**C**(O)CH_2_CH_3_); HRMS (ESI): *m*/*z* calcd for C_21_H_24_O_4_ [M+Na]^+^: 363.1567; found: 363.1571; IR (ATR): ν_max_ = 1728, 1508, 1183, 1136, 996, 957, 750, 697 cm^−1^.

#### 3.4.3. Preparative CALB-Catalyzed Transesterification of Alcohol **1**

CALB (0.9 g) was added to a solution of racemic alcohol **1** (1.8 g, 6.34 mmol) and vinyl propionate (1 mL, 9.89 mmol) in 90 mL of DIPE. The reaction mixture was stirred in a 250 mL round-bottomed flask at room temperature. After 6 h, the enzyme was removed by filtration and the organic solvent was evaporated. The enantiomerically enriched products were isolated by flash chromatography with a gradient elution from 100% *n*-hexane to a *n*-hexane/ethyl acetate 3:1, *v*/*v*.

*(−)-(S,E)-4-(4′-Benzyloxy-3′-methoxyphenyl)but-3-en-2-ol ((S)-* **1** *)*

Yield, 0.87 g (48%); white solid; ee = 98%; *t*_R_ = 34.2 min; ${\left[\alpha \right]}_{D}^{20}$ = −30.6 (*c* 1.0, CH_2_Cl_2_). The physical and spectroscopic data were consistent with those reported for racemic alcohol **1** [33].

*(+)-(R,E)-4-(4′-Benzyloxy-3′-methoxyphenyl)but-3-en-2-yl propionate ((R)-* **3** *)*

Yield, 1.13 g (52%); white crystals; ee = 85%; *t*_R_ = 11.6 min; ${\left[\alpha \right]}_{D}^{20}$ = +21.9 (*c* 1.05, CH_2_Cl_2_). The physical and spectroscopic data were consistent with those reported herein for the racemic propionate **3**.

#### 3.4.4. Preparation of (*S*)-Zingerol for Configuration Assignment

Zingerol was obtained by catalytic hydrogenation of alcohol (*S*)-**1** according to the procedure reported by Enders et al. [49], with minor modifications. Alcohol (*S*)-**1** (0.26 g, 0.91 mmol) was dissolved in 12 mL of UHPLC-grade methanol, and 0.38 g of 10% Pd/C was added. The reaction mixture was stirred at room temperature under a hydrogen atmosphere for 48 h. The progress of the reaction was monitored by TLC. After the completion of the reaction, the catalyst was removed by filtration, and the solvent was evaporated under reduced pressure. The crude product was isolated by flash chromatography with a gradient elution from 100% *n*-hexane to a *n*-hexane/ethyl acetate 2.5:1, *v*/*v* to afford (*S*)-zingerol. Physical and spectroscopic data for the synthesized (*S*)-zingerol are provided below:

*(+)-(S)-4-(4′-hydroxy-3′-methoxyphenyl)butan-2-ol (**(S)-zingerol***)

Yield, 0.09 g (50%); colorless oily liquid; ${\left[\alpha \right]}_{D}^{20}$ = +13.2 (*c* 0.98, EtOH) (lit. +14.1 (*c* 0.91, EtOH) [38]); ^1^H NMR (600 MHz, CDCl_3_): δ 1.23 (d, *J* = 6.2 Hz, 3H, C**H_3_**-1), 1.46 (br s, 1H, CH-O**H**), 1.71–1.79 (m, 2H, CH_2_-3), 2.60 (ddd, *J* = 13.9, 9.3 and 6.9 Hz, 1H, one of CH_2_-4), 2.69 (ddd, *J* = 13.9, 9.5 and 6.1 Hz, 1H, one of CH_2_-4), 3.83 (m, 1H, H-2), 3.87 (s, 3H, -OC**H_3_**), 5.54 (s, 1H, Ar-O**H**), 6.69 (dd, *J =* 7.9 and 1.8 Hz, 1H, H-6′), 6.70 (d, *J* = 1.8 Hz, 1H, H-2′), 6.83 (d, *J* = 7.9 Hz, 1H, H-5′); ^13^C NMR (150 MHz, CDCl_3_): δ 23.62 (C-1), 31.80 (C-4), 41.09 (C-3), 55.84 (-O**C**H_3_), 67.53 (C-2), 110.97 (C-2′), 114.25 (C-5′), 120.85 (C-6′), 133.94 (C-1′), 143.67 (C-4′), 146.41 (C-3′).

#### 3.4.5. Hydrolysis of Propionate (*R*)-**3**

Propionate (*R*)-**3** (1.13 g, 3.32 mmol) was heated at reflux in 120 mL of a 6% NaOH solution in ethanol. When the substrate was completely hydrolyzed (3.5 h, TLC), the ethanol was removed under reduced pressure and the residue was diluted with distilled water. The product was extracted with dichloromethane (3 × 30 mL). The combined organic layers were washed with brine until neutral and dried over anhydrous MgSO_4_. Evaporation of the solvent under reduced pressure afforded crude (*R*)-**1**, which was subsequently recrystallized from *n*-hexane to give pure (*R*)-**1**.

*(+)-(R,E)-4-(4′-Benzyloxy-3′-methoxyphenyl)but-3-en-2-ol ((R)-* **1** *)*

Yield, 0.91 g (96%); white solid; ee = 89% (after recrystallization from *n*-hexane); *t*_R_ = 41.1 min; ${\left[\alpha \right]}_{D}^{20}$ = +28.8 (*c* 1.05, CH_2_Cl_2_). The physical and spectroscopic data were consistent with those previously reported for the racemic alcohol **1** [33].

### 3.5. Synthesis of Enantiomerically Enriched Iodolactones ***7a**–**c***

#### 3.5.1. Johnson–Claisen Rearrangement

Enantiomeric alcohols (*S*)-**1** and (*R*)-**1** were subjected to the Johnson–Claisen rearrangement by reaction with triethyl orthoacetate at 138 °C for 6 h. The details of synthesis, purification, and spectroscopic data for both obtained enantiomers were consistent with those previously reported for the racemic ester **4** [33].

*(−)-(R,E)-3-(4′-benzyloxy-3′-methoxyphenyl)hex-4-enoate ((R)-* **4** *)*

Obtained from 0.87 g (3.07 mmol) of alcohol (*R*)**-1**; yield, 0.41 g (38%); pale-yellow oily liquid; ee = 76%, *t*_R_ = 9.1 min; ${\left[\alpha \right]}_{D}^{20}$ = −6.5 (*c* 1.0, CH_2_Cl_2_).

*(+)-(S,E)-3-(4′-benzyloxy-3′-methoxyphenyl)hex-4-enoate ((S)-* **4** *)*

Obtained from 0.87 g (3.07 mmol) of alcohol (*S*)**-1**; yield, 0.37 g (34%); pale-yellow oily liquid; ee = 95%; *t*_R_ = 9.6 min; ${\left[\alpha \right]}_{D}^{20}$ = +7.8 (*c* 1.0, CH_2_Cl_2_).

#### 3.5.2. Hydrolysis of γ,δ-Unsaturated Esters (*R*)-**4** and (*S*)-**4**

Esters *(R)*-**4** and (*S*)-**4** were hydrolyzed in 5% ethanolic solution of NaOH. The reaction protocol and the physical and spectroscopic data of both obtained enantiomers (*R*)-**5** and (*S*)-**5** were consistent with those reported previously for the racemic form [33].

*(−)-(R,E)-3-(4′-Benzyloxy-3′-methoxyphenyl)hex-4-enoic acid ((R)-* **5** *)*

Obtained from 0.37 g (1.05 mmol) of ester (*R*)-**4**; yield, 0.29 g (85%); yellow liquid; ee = 81%; *t*_R_ = 28.0 min; ${\left[\alpha \right]}_{D}^{20}$ = −1.2 (*c* 1.0, CH_2_Cl_2_).

*(+)-(S,E)-3-(4′-Benzyloxy-3′-methoxyphenyl)hex-4-enoic acid ((S)-* **5** *)*

Obtained from 0.37 g (1.05 mmol) of ester (*S*)-**4**; yield, 0.31 g (90%); yellow liquid; ee = 94%; *t*_R_ = 25.9 min; ${\left[\alpha \right]}_{D}^{20}$ = +1.4 (*c* 1.0, CH_2_Cl_2_).

#### 3.5.3. Iodolactonization of Acids (*R*)-**5** and (*S*)-**5**

Acids (*R*)-**5** and (*S*)-**5** were subjected to iodolactonization using iodine in NaHCO_3_/Et_2_O biphasic system. The reaction conditions, purification and procedures, physical and spectroscopic data of the isolated enantiomerically enriched iodolactones **6a**–**c** were consistent with those previously reported for their racemic counterparts [33].

After iodolactonization of acid (*R*)-**5** (0.29 g, 0.89 mmol), the following products were isolated:

*(−)-cis-(4S,5S,6R)-4-(4′-benzyloxy-3′-methoxyphenyl)-5-(1-iodoethyl)dihydrofuran-2-one ((4S,5S,6R)-***6a***)*

Yield, 0.19 g (48%); white solid; ee = 81%; *t*_R_ = 27.6 min; ${\left[\alpha \right]}_{D}^{20}$ = −3.9 (*c* 1.0, CH_2_Cl_2_).

*(−)-trans-(4S,5R,6S)-4-(4′-Benzyloxy-3′-methoxyphenyl)-5-(1-iodoethyl)dihydrofuran-2-one ((4S,5R,6S)-* **6b** *)*

Yield, 0.10 g (24%); white solid; ee = 64%; *t*_R_ = 38.6 min; ${\left[\alpha \right]}_{D}^{20}$ = −6.8 (*c* 1.0, CH_2_Cl_2_).

*(+)-(4S,5S,6R)-4-r-(4′-Benzyloxy-3′-methoxyphenyl)-5-t-iodo-6-c-methyltetrahydropyran-2-one ((4S,5S,6R)-* **6c** *)*

Yield, 0.04 g (10%); white solid; ee >99%; *t*_R_ = 88.4 min; ${\left[\alpha \right]}_{D}^{20}$ = +20.1 (*c* 0.7, CH_2_Cl_2_).

After iodolactonization of acid (*S*)-**5** (0.29 g, 0.89 mmol), the following products were isolated:

*(+)-cis-(4R,5R,6S)-4-(4′-Benzyloxy-3′-methoxyphenyl)-5-(1-iodoethyl)dihydrofuran-2-one ((4R,5R,6S)-***6a***)*

Yield, 0.16 g (40%); white solid; ee = 85%; *t*_R_ = 19.5 min; ${\left[\alpha \right]}_{D}^{20}$ = +4.0 (*c* 1.0, CH_2_Cl_2_).

*(+)-trans-(4R,5S,6R)-4-(4′-Benzyloxy-3′-methoxyphenyl)-5-(1-iodoethyl)dihydrofuran-2-one ((4R,5S,6R)-* **6b** *)*

Yield, 0.07 g (18%); white solid; ee = 74%; *t*_R_ = 33.1 min; ${\left[\alpha \right]}_{D}^{20}$ = +9.1 (*c* 1.0, CH_2_Cl_2_).

*(−)-(4R,5R,6S)-4-r-(4′-Benzyloxy-3′-methoxyphenyl)-5-t-iodo-6-c-methyltetrahydropyran-2-one ((4R,5R,6S)-* **6c** *)*

Yield, 0.02 g (4%); white solid; ee >99%; *t*_R_ = 86.4 min; ${\left[\alpha \right]}_{D}^{20}$ = −20.2 (*c* 1.0, CH_2_Cl_2_).

#### 3.5.4. General Procedure for Benzyl Deprotection of Enantiomerically Enriched Iodolactones **6a**,**b**

Enantiomerically enriched iodolactones **6a**,**b** (0.07 g, 0.16 mmol) were dissolved in anhydrous dichloromethane (10 mL) in a two-necked flask equipped with a septum and a thermometer. The solution was cooled to −68 °C using a cryostat with technical-grade ethanol, and 1 M BCl_3_ in dichloromethane (13.5 mL, 13.5 mmol) was added dropwise via syringe under stirring. After 20 min, the reaction was quenched with 25 mL of 3% HCl and 40 mL of distilled water, and the product was extracted with dichloromethane (3 × 20 mL). The combined organic layers were washed with brine and dried over anhydrous MgSO_4_. The solvent was removed under reduced pressure using a rotary evaporator and the concentrated crude product was purified by flash chromatography. Gradient elution from 100% *n*-hexane to *n*-hexane/ethyl acetate 9:2 (*v*/*v*) was used for lactones **7a**, while a gradient from 100% *n*-hexane to *n*-hexane/ethyl acetate 3:1 (*v*/*v*) was applied for lactones **7b**. The lactones **7a** and **7b** obtained after flash chromatography were subsequently recrystallized from *n*-hexane to afford the corresponding enantiomerically enriched iodolactones. The physical and spectroscopic data of the deprotected lactones were consistent with those published previously for the corresponding racemic counterparts [33]. The reported ee values refer to the recrystallized products.

*(−)-cis-(4S,5S,6R)-4-(4′-Hydroxy-3′-methoxyphenyl)-5-(1-iodoethyl)dihydrofuran-2-one ((4S,5S,6R)-* **7a** *)*

Obtained from 0.07 g (0.16 mmol) of iodolactone (*4S*,*5S*,*6R*)-**6a**; yield, 0.03 g (60%); white solid; ee = 86%; *t*_R_ = 35.7 min; ${\left[\alpha \right]}_{D}^{20}$ = −16.8 (*c* 1.5, CH_2_Cl_2_).

*(+)-cis-(4R,5R,6S)-4-(4′-Hydroxy-3′-methoxyphenyl)-5-(1-iodoethyl)dihydrofuran-2-one ((4R,5R,6S)-* **7a** *)*

Obtained from 0.07 g (0.16 mmol) of iodolactone (*4R*,*5R*,*6S*)-**6a**; yield, 0.04 g (76%); white solid; ee = 97%; *t*_R_ = 24.5 min; ${\left[\alpha \right]}_{D}^{20}$ = +18.9 (*c* 1.7, CH_2_Cl_2_).

*(−)-trans-(4S,5R,6S)-4-(4′-Hydroxy-3′-methoxyphenyl)-5-(1-iodoethyl)dihydrofuran-2-one ((4S,5R,6S)-* **7b** *)*

Obtained from 0.07 g (0.16 mmol) of iodolactone (*4S*,*5R*,*6S*)-**6b**; yield, 0.04 g (66%); white solid; ee = 64%; *t*_R_ = 47.1 min; ${\left[\alpha \right]}_{D}^{20}$ = −8.9 (*c* 1.3, CH_2_Cl_2_).

*(+)-trans-(4R,5S,6R)-4-(4′-Hydroxy-3′-methoxyphenyl)-5-(1-iodoethyl)dihydrofuran-2-one ((4R,5S,6R)-* **7b** *)*

Obtained from 0.07 g (0.16 mmol) of iodolactone (*4R*,*5S*,*6R*)-**6b**; yield, 0.04 g (67%); white solid; ee = 97%; *t*_R_ = 41.0 min; ${\left[\alpha \right]}_{D}^{20}$ = +12.3 (*c* 2.2, CH_2_Cl_2_).

### 3.6. MTT Assay

Cell viability was assessed using the MTT assay previously described by Dunal et al. [34]. Briefly, the cell line LM-MEL-75 (human melanoma) was purchased from the American Type Culture Collection (Rockville, MD, USA). The cell lines A2780 (human ovarian carcinoma) and EPG85-257RDB (human gastric adenocarcinoma) were kindly provided by Associate Professor T. Gębarowski, Division of Animal Anatomy, Department of Biostructure and Animal Physiology, Wrocław University of Environmental and Life Sciences, Poland. Normal human dermal fibroblasts (NHDFs), used as a normal cell line, were purchased from PromoCell (Heidelberg, Germany). The cell lines used in this study were authenticated and certified as mycoplasma-free by the respective vendors prior to distribution. Cells were cultured under standard conditions at 37 °C in a humidified atmosphere containing 5% CO_2_ (EuroClone, Pero, Italy). NHDF cells were cultured in DMEM, whereas LM-MEL-75, A2780, and EPG85-257RDB cells were cultured in RPMI-1640 medium. Both media were supplemented with 10% FBS.

Cells of each cell line were seeded at a density of 5 × 10^3^ cells per well in 96-well plates (Eppendorf, Hamburg, Germany) in 100 µL of culture medium per well and allowed to attach for 24 h prior to treatment. Subsequently, 100 µL of the working solutions of the tested compounds, prepared by appropriate dilution of the corresponding DMSO stock solutions with culture medium, was added to each well, resulting in a final volume of 200 µL per well. The tested enantiomerically enriched iodolactones **7a**,**b** and their racemic counterparts, *rac*-**7a** and *rac*-**7b,** were applied at concentrations ranging from 1.38 to 55.25 µM and incubated for 24 h. Cell viability was measured using the MTT assay, which quantifies the enzymatic reduction of tetrazolium salts by metabolically active cells. For the MTT assay, MTT was added to each well at a final concentration of 1 mg/mL and cells were incubated for 2 h at 37 °C. Following incubation, the formed formazan crystals were dissolved by adding 100 µL of isopropanol to each well.

Absorbance was measured spectrophotometrically at 570 nm (signal) and 630 nm (reference) using a Multiskan GO microplate spectrophotometer (Thermo Fisher Scientific, Waltham, MA, USA). The absorbance measured at 630 nm was subtracted from that measured at 570 nm (A570 − A630), and background absorbance determined in cell-free wells containing culture medium and MTT reagent was subsequently subtracted from the corresponding corrected values before the calculation of cell viability. The final DMSO concentration in the culture wells did not exceed 0.1% (*v*/*v*), a level generally considered non-toxic to cells [50]; therefore, a separate vehicle control was not applied. Untreated cells served as the negative control, whereas doxorubicin-treated cells were used as the positive control. Cell viability was normalized to the untreated control, defined as 100%, and the viability of treated cells was expressed as a percentage of the untreated control.

IC_50_ values were determined from the concentration–response data using the Quest Graph™ IC50 Calculator (AAT Bioquest, Inc., Pleasanton, CA, USA) by nonlinear regression based on a four-parameter logistic (4PL) model. Experimental concentration values were entered directly into the calculator without prior manual log transformation. The minimum and maximum responses were estimated as model parameters, and the upper and lower plateaus were not manually constrained. When 50% inhibition was not reached within the tested concentration range, the IC_50_ value was reported as greater than the highest tested concentration (>55.25 µM), without extrapolation beyond the experimental concentration range. IC_50_ values were determined separately for each of the four independent biological experiments. Four technical replicates (wells) were performed per biological replicate. The resulting four IC_50_ values were subsequently used to calculate the mean and standard deviation (SD), which are reported as the final IC_50_ values (mean ± SD, n = 4). Data from independent biological experiments were not pooled into a single fitted concentration–response curve.

### 3.7. TUNEL Assay

The ApopTag^®^ Peroxidase In Situ Apoptosis Detection Kit (Merck Millipore, Billerica, MA, USA) was used in this study. The kit contained equilibration buffer, working-strength TdT enzyme, Stop/Wash buffer, anti-digoxigenin peroxidase conjugate, and DAB peroxidase substrate. EPG85-257RDB cells were seeded onto 10-well hydrophobic microscope slides (Thermo Fisher Scientific, Waltham, MA, USA) at a density of 2 × 10^4^ cells in 40 μL of RPMI-1640 medium with 10% FBS, L-glutamine (4 mM), penicillin (100 U/mL) and streptomycin (100 μg/mL) per well and incubated for 24 h. After attachment, the culture medium was replaced and the cells were exposed to selected concentrations of the tested compound (4*R*,5*S*,6*R*)-**7b** for another 24 h (0.5, 1, 2, 5, 10 and 20 µg/mL, corresponding to 1.38, 2.76, 5.52, 13.81, 27.62, 55.25 µM; molecular weight (*M*_W_), used to recalculate the concentrations, was 362 g/mol for lactone **7b**. The treatment solutions were prepared by appropriate dilution of the corresponding compound stock solution in DMSO with the culture medium. The final DMSO concentration in the culture wells did not exceed 0.1% (*v*/*v*); therefore, a separate vehicle control was not included, and untreated cells served as negative controls.

**Figure 2:**
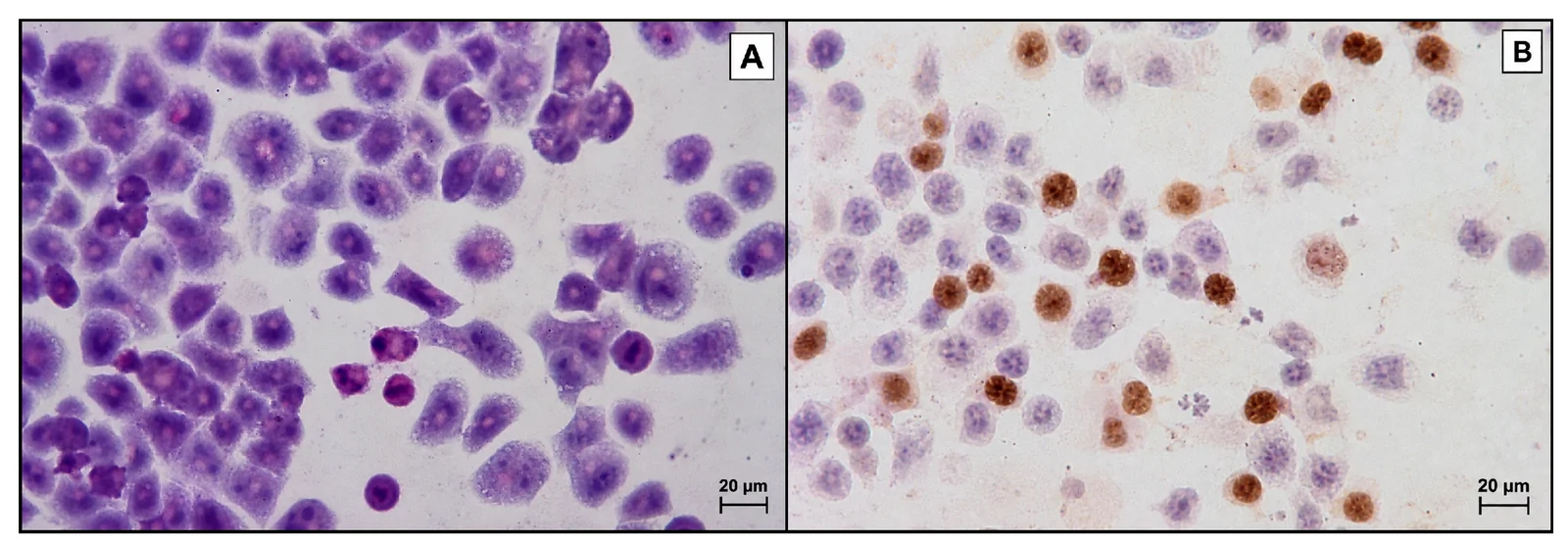
Representative images of positive and negative controls used for the TUNEL assay. (**A**) Negative labeling control performed without TdT enzyme, showing the absence of specific brown nuclear staining. (**B**) Positive control treated with DNase I prior to TUNEL labeling, showing strong brown nuclear staining indicative of DNA fragmentation. Scale bar = 20 µm.

After treatment, the cells were fixed in 1% paraformaldehyde (pH 7.4) for 10 min at room temperature. The slides were then washed twice with PBS for 5 min each time and then incubated in a pre-chilled 1:1 mixture of ethanol and acetic acid for 5 min at −20 °C to ensure cell permeabilization and attachment to the slide surface. After two additional washes with PBS, endogenous peroxidase activity was quenched by incubation in 3% hydrogen peroxide for 5 min. In addition, a separate positive control for DNA fragmentation was prepared from untreated cells by treatment with DNase I prior to TUNEL labeling to induce DNA strand breaks and generate free 3′-OH termini. The positive-control samples were subsequently subjected to the same TdT-mediated labeling and DAB detection procedure as the experimental samples. A strong brown nuclear signal in the positive-control samples confirmed the proper performance of the TUNEL labeling and detection procedure. A negative labeling control (untreated cells) was also included by processing a separate sample through the same TUNEL procedure while omitting the TdT enzyme during the labeling step. The negative control sample was subsequently subjected to the same washing, anti-digoxigenin peroxidase conjugation, and DAB detection steps as the experimental samples, followed by hematoxylin and eosin (H&E) counterstaining. The absence of specific brown nuclear staining in the negative control sample confirmed that the observed TUNEL signal was dependent on TdT-mediated labelling (Figure 2).

The samples were washed again with PBS and immediately covered with equilibration buffer (75 μL/5 cm^2^) for approximately 1 min. After removing excess buffer, working-strength TdT enzyme (55 μL/5 cm^2^) was added to each sample and the slides were incubated in a humid chamber at 37 °C for 1 h. The reaction was terminated by immersing the slides in Stop/Wash buffer for 10 min at room temperature, followed by three washes with PBS (1 min each). Anti-digoxigenin peroxidase conjugate (65 μL/5 cm^2^) was then added and the samples were incubated in a humid chamber for 30 min at room temperature. After four washes in PBS (2 min each), DAB peroxidase substrate (75 μL/5 cm^2^) was applied for 10 min to visualize TUNEL-positive cells. The slides were washed three times with double-distilled water and incubated in fresh double-distilled water for another 5 min. Cell nuclei were counterstained with hematoxylin and eosin (H&E). Finally, samples were dehydrated by immersion in 70% ethanol for 30 s and then in xylene for 30 s. Coverslips were mounted using glass mounting medium (Thermo Fisher Scientific, Waltham, MA, USA).

The percentage of TUNEL-positive cells was determined in five randomly selected microscopic fields at 400× magnification using a Zeiss Axio Scope A1 light microscope (Carl Zeiss, Jena, Germany). For each field, the total cell population was counted and defined as 100%, and the proportion of TUNEL-positive cells was subsequently calculated. The results were expressed as the percentage of TUNEL-positive cells relative to the total number of cells. Results are presented as mean values obtained from five microscopic fields and four independent experiments. Approximately 100 cells were evaluated per microscopic field, corresponding to approximately 500 cells per biological replicate across the five randomly selected fields. All evaluations were performed independently by two experienced observers who were blinded to the treatment conditions and were trained in the assessment of histological and immunohistochemical specimens.

### 3.8. Cell Cycle Analysis

Cell cycle distribution was evaluated by flow cytometric analysis of DNA content using propidium iodide staining. EPG85-257RDB cells were seeded 24 h before the experiment in 6-well plates (Eppendorf, Hamburg, Germany) and exposed to the test compound (4*R*,5*S*,6*R*)-**7b** at selected concentrations (0.5, 1, 2, 5, 10 and 20 µg/mL, corresponding to 1.38, 2.76, 5.52, 13.81, 27.62, and 55.25 µM; *M*_W_, used to recalculate the concentrations, was 362 g/mol for lactone **7b** for 24 h. The treatment solutions were prepared by the appropriate dilution of the corresponding compound stock solution in DMSO with the culture medium. The final DMSO concentration in the culture wells did not exceed 0.1% (*v*/*v*); therefore, a separate vehicle control was not included, and untreated cells served as the negative controls.

After incubation, cells were washed with PBS, detached with trypsin (0.25%)–EDTA (0.02%) and resuspended in 1 mL RPMI-1640 medium with 10% FBS, L-glutamine (4 mM), penicillin (100 U/mL) and streptomycin (100 μg/mL). Harvested cells were centrifuged at 1200 rpm for 5 min, after which the culture medium was removed and the cells were resuspended in 1 mL of PBS. Cells were then counted and a total of 1 × 10^6^ cells were transferred to Eppendorf tubes (Eppendorf, Hamburg, Germany) and resuspended in 1 mL of PBS. The cell suspension was centrifuged at 1200 rpm for 5 min at 4 °C. After removing the supernatant, the cell pellet was resuspended in 0.3 mL of PBS. Ice-cold 70% ethanol (0.7 mL) was added dropwise with gentle mixing to prevent cell aggregation. The resulting suspension was incubated on ice for 1 h.

After fixation, the cells were centrifuged under the same conditions as described above. The cell pellet was washed once with PBS and centrifuged again. After removing the supernatant, the cells were resuspended in 0.25 mL of PBS and 5 μL of RNase solution was added. The samples were incubated for 1 h at 37 °C. Then, 10 μL of propidium iodide solution (1 mg/mL) was added. Stained cells were transferred to flow cytometry tubes and analyzed using a Muse^®^ Cell Analyzer (Cytek Biosciences, Fremont, CA, USA) equipped with a 488 nm laser. For each sample, 5000 events were acquired using the default acquisition settings of the Muse^®^ Cell Cycle assay. Cell cycle analysis was performed using the Cell Cycle assay module. The flow cytometry analysis was not performed under blinded conditions. The cellular population of interest was identified and gated on the DNA Content Index versus Cell Size Index plot. Cellular debris was excluded by adjustment of the DNA content threshold according to the manufacturer’s recommendations, while events outside the defined analysis region, including high-DNA-content cell aggregates, were excluded from cell cycle phase quantification. Cell cycle distribution was subsequently evaluated from DNA content histograms, with populations assigned to the G0/G1, S, and G2/M phases according to their DNA content. Floating and spontaneously detached cells present in the culture medium were not included in the analysis; therefore, the cell cycle analysis was restricted to the adherent cell population. All experiments were performed in four independent biological replicates. The percentages of cells in the G0/G1, S, and G2/M phases were determined from the DNA content histograms using Flowing Software (version 2.5.1, Turku, Finland).

### 3.9. Statistical Analysis

Analyses were performed using GraphPad Prism 8 software (GraphPad Software, San Diego, CA, USA). Prior to hypothesis testing, data distribution normality was assessed. Statistical analyses were performed using one-way analysis of variance (ANOVA). Depending on the experimental design, post hoc analyses were performed using Dunnett’s multiple comparison test for comparisons with the control group or Tukey’s multiple comparison test for pairwise comparisons among the treated groups.

## 4. Conclusions

The results indicate that, under the experimental conditions used, no statistically significant differences in cell-viability-reducing activity were observed among the tested enantiomerically enriched forms of the vanillin-derived δ-iodo-γ-lactones. Instead, the observed activity was more closely associated with the *cis*/*trans* stereochemistry of the γ-lactone ring, with the *trans* isomers generally exhibiting substantially higher activity than their *cis* counterparts. Notably, the (4*R*,5*S*,6*R*)-enantiomer of *trans* δ-iodo-γ-lactone **7b** was identified as the most active derivative, showing pronounced cell-viability-reducing effects and a high selectivity index, particularly against the multidrug-resistant gastric cancer cell line EPG85-257RDB.

The cellular effects of (4*R*,5*S*,6*R*)-**7b** were further characterized, providing preliminary evidence that treatment with this compound is associated with concentration-dependent DNA fragmentation and alterations in cell cycle distribution in EPG85-257RDB cells, which may contribute to the reduced cancer cell viability observed in the MTT assay. The observed cell-viability-reducing activity of (4*R*,5*S*,6*R*)-**7b** in EPG85-257RDB cells, together with its lower short-term cytotoxicity against normal NHDFs than toward the tested cancer cell lines, highlights (4*R*,5*S*,6*R*)-**7b** as a promising lead compound for further mechanistic and formulation studies. However, the present selectivity assessment was limited to a single non-malignant cell model and a 24 h exposure period; therefore, no conclusions regarding systemic safety can be drawn from these data. Additional studies using matched drug-sensitive and other drug-resistant cell lines are required to determine whether multidrug resistance influences its cell-viability-reducing activity.

Further mechanistic studies will also include comparative evaluations of less active or inactive stereoisomers and investigations of apoptosis-related and cell-cycle-regulatory pathways (Bcl-2 family proteins, DNA damage markers) to determine whether the observed effects are specifically associated with the cell-viability-reducing activity of (4*R*,5*S*,6*R*)-**7b**. Such studies would help to distinguish activity-related effects from nonspecific cellular toxicity.

Longer-term studies will focus on the development of phospholipid-based liposomal nanocarriers incorporating iodolactone **7b** to improve their physicochemical stability, bioavailability and delivery efficiency. These studies will include characterization of the physicochemical properties of the liposomal systems, evaluation of the encapsulation efficiency, assessment of nanosystem stability, and detailed studies of the interactions between iodolactone-loaded liposomes and both modelled and biological membranes.

## Figures and Tables

**Table 1:** Results of the enzymatic transesterification of racemic allylic alcohol **1** using different acyl donors.

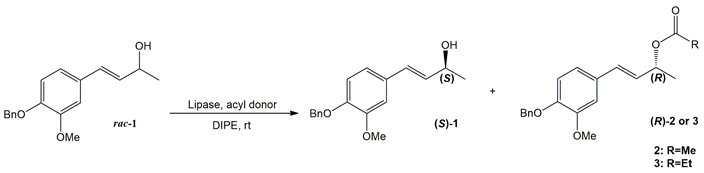							
Entry	Enzyme	Acyl Donor	Time [h]	Conversion ^a^ [mol%]	ee of (*S*)-Alcohol 1 [%]	ee of (*R*)-Ester 2 or 3 [%]	E ^b^
**1**	Amano Lipase PS	Isopropenyl acetate	24	34	43	93	44
**2**		Vinyl propionate	24	46	71	99	>100
**3**	Lipozyme^®^	Isopropenyl acetate	24	12	12	88	18
**4**		Vinyl propionate	24	53	95	99	>100
**5**	CALB	Isopropenyl acetate	6	54	99	93	>100
**6**		Vinyl propionate	6	54	99	99	>100

^a^ According to chiral HPLC analysis. ^b^ The enantiomeric ratio calculated at the highest conversion rate according to the following equation: E = ln[(1 − ee_s_)/(1 + (ee_s_/ee_e_)]/ln[(1 + ee_s_)/(1 + (ee_s_/ee_e_)]. ee_s_ = enantiomeric excess of unreacted alcohol; ee_e_ = enantiomeric excess of acetate or propionate.

**Table 2:** Effects of enantiomerically enriched *cis*-δ-iodo-γ-lactones **7a** and *trans*-δ-iodo-γ-lactones **7b** on the viability of LM-MEL-75, EPG85-257RDB, A2780, and NHDF cell lines after 24 h of treatment.

Compound	Cell Line			
	LM-MEL-75	EPG85-257RDB	A2780	NHDF
	IC_50_ [µM] ^1^			
*rac*-**7a**	30.77 ± 3.07 ^a^	35.88 ± 9.94 ^a^	26.85 ± 7.04 ^a^	51.76 ± 9.83 ^ab^
*cis*-(4*S*,5*S*,6*R*)-**7a**	28.84 ± 4.78 ^a^	28.88 ± 10.28 ^abc^	24.50 ± 3.81 ^abc^	45.66 ± 7.82 ^ab^
*cis*-(4*R*,5*R*,6*S*)-**7a**	27.15 ± 1.33 ^a^	20.77 ± 9.61 ^ab^	18.15 ± 10.28 ^ab^	41.27 ± 11.85 ^ab^
*rac*-**7b**	12.04 ± 4.78 ^b^	6.24 ± 3.65 ^cd^	14.17 ± 6.82 ^abcd^	>55.25 ^a^
*trans*-(4*S*,5*R*,6*S*)-**7b**	9.89 ± 3.45 ^b^	5.08 ± 1.30 ^d^	9.06 ± 5.36 ^bcd^	51.74 ± 9.06 ^b^
*trans*-(4*R*,5*S*,6*R*)-**7b**	6.85 ± 7.79 ^b^	3.98 ± 2.60 ^d^	11.80 ± 1.08 ^cd^	36.69 ± 8.81 ^ab^
Doxorubicin	2.18 ± 1.37 ^b^	5.48 ± 0.75 ^bcd^	1.30 ± 1.17 ^d^	0.44 ± 0.31 ^c^

^1^ Data represent mean values ± SD from four independent biological replicates (n = 4), with four technical replicates (wells) per biological replicate. Statistical analysis was performed using one-way analysis of variance (ANOVA) followed by Tukey’s multiple comparison test for pairwise comparisons among groups. Analyses were performed separately within each cell line. Values not sharing a common superscript letter (a, b, c, d) within the same column were considered significantly different at *p* < 0.05.

**Table 3:** SI for enantiomerically enriched *cis*-δ-iodo-γ-lactones **7a** and *trans*-δ-iodo-γ-lactones **7b** against LM-MEL-75, EPG85-257RDB and A2780 cell lines.

Compound	Cell Line		
	LM-MEL-75	EPG85-257RDB	A2780
	SI ^1^		
*cis*-(4*S*,5*S*,6*R*)-**7a**	1.58	1.58	1.86
*cis*-(4*R*,5*R*,6*S*)-**7a**	1.52	1.99	2.27
*trans*-(4*S*,5*R*,6*S*)-**7b**	5.23	10.19	5.71
*trans*-(4*R*,5*S*,6*R*)-**7b**	5.36	9.22	3.11
Doxorubicin	0.20	0.08	0.34

^1^ SI calculated as the ratio of the IC_50_ value obtained for the normal cell line (NHDF) to that measured for the corresponding cancer cell line.

**Table 4:** Percentage of TUNEL-positive cells in the EPG85-257RDB cell line following 24 h of treatment with iodolactone (4*R*,5*S*,6*R*)-**7b** at concentrations ranging from 0.5 to 20 µg/mL, corresponding to 1.38–55.25 µM.

Concentration [μg/mL]	TUNEL-Positive Cells [%] ^1^
Untreated control	3.05 ± 0.29 ^f^
0.5	8.30 ± 0.95 ^ef^
1	12.70 ± 1.42 ^e^
2	19.60 ± 1.90 ^d^
5	35.80 ± 2.73 ^c^
10	52.40 ± 3.37 ^b^
20	74.10 ± 4.26 ^a^

^1^ Data represent mean values ± SD from four independent biological replicates (n = 4); no technical replicates were performed. Statistical analysis was performed using ANOVA followed by Tukey’s multiple comparison test for pairwise comparisons among groups. Values not sharing a common superscript letter (a, b, c, d, e, f) within the same column were considered significantly different at *p* < 0.05.

## Data Availability

The data presented in this study are available in the Supplementary Material of this article.

## Supplementary Materials

The following supporting information can be downloaded at: https://www.mdpi.com/article/10.3390/ijms27177928/s1.
