# Supplementary material for: From Chemoenzymatic Synthesis to Biological Effects: Enantiomerically Enriched Vanillin-Derived δ-Iodo-γ-Lactones as Modulators of Cancer Cell Viability and Inducers of Cell Death
Source: Int J Mol Sci. 2026 Sep 5;27(17):7928. doi: 10.3390/ijms27177928 (PMC13565921; doi:10.3390/ijms27177928)
Supplement: Supplementary file 1 [file ijms-27-07928-s001.zip › ijms-4490356-supplementary.pdf]

Supplementary Information

From Chemoenzymatic Synthesis to Biological Effects: Enantiomerically Enriched Vanillin-Derived δ-Iodo-γ-Lactones as Modulators of Cancer Cell Viability and Inducers of Cell Death

Anna Dunal[1,*], Dominik Poradowski[2], Stefano Serra[3], Aleksander Chrószcz[2], Hanna Pruchnik[4] and Witold Gładkowski[1,*]

1 Department of Food Chemistry and Biocatalysis, Wrocław University of Environmental and Life Sciences, Norwida 25, 50-375 Wrocław, Poland

2 Department of Biostructure and Animal Physiology, Division of Animal Anatomy, Faculty of Veterinary Medicine, Wrocław University of Environmental and Life Sciences, Kożuchowska 1, 51-631 Wrocław, Poland; dominik.poradowski@upwr.edu.pl (D.P.); aleksander.chroszcz@upwr.edu.pl (A.C.)

3 Consiglio Nazionale delle Ricerche (C.N.R.) Istituto di Scienze e Tecnologie Chimiche, Via Mancinelli 7, 20131 Milano, Italy; stefano.serra@cnr.it

4 Department of Physics and Biophysics, Faculty of Biotechnology and Food Sciences, Wrocław University of Environmental and Life Sciences, Norwida 25, 50-375 Wrocław, Poland; hanna.pruchnik@upwr.edu.pl

* Correspondence: anna.dunal@upwr.edu.pl (A.D.); witold.gladkowski@upwr.edu.pl (W.G.)

Table of content

	Pages
**Figure S1.** ^{1}H NMR spectrum of (*E*)-4-(4'-benzyloxy-3'-methoxyphenyl)but-3-en-2-yl acetate (**2**)	4
**Figure S2.** ^{13}C NMR spectrum of (*E*)-4-(4'-benzyloxy-3'-methoxyphenyl)but-3-en-2-yl acetate (**2**)	4
**Figure S3.** DEPT 135 NMR spectrum of (*E*)-4-(4'-benzyloxy-3'-methoxyphenyl)but-3-en-2-yl acetate (**2**)	5
**Figure S4.** COSY spectrum of (*E*)-4-(4'-benzyloxy-3'-methoxyphenyl)but-3-en-2-yl acetate (**2**)	5
**Figure S5.** HMQC spectrum of (*E*)-4-(4'-benzyloxy-3'-methoxyphenyl)but-3-en-2-yl acetate (**2**)	6
**Figure S6.** HMBC spectrum of (*E*)-4-(4'-benzyloxy-3'-methoxyphenyl)but-3-en-2-yl acetate (**2**)	6
**Figure S7.** ^{1}H NMR spectrum of (*E*)-4-(4'-benzyloxy-3'-methoxyphenyl)but-3-en-2-yl propionate (**3**)	7
**Figure S8.** ^{13}C NMR spectrum of (*E*)-4-(4'-benzyloxy-3'-methoxyphenyl)but-3-en-2-yl propionate (**3**)	7
**Figure S9.** DEPT 135 NMR spectrum of (*E*)-4-(4'-benzyloxy-3'-methoxyphenyl)but-3-en-2-yl propionate (**3**)	8
**Figure S10.** COSY spectrum of (*E*)-4-(4'-benzyloxy-3'-methoxyphenyl)but-3-en-2-yl propionate (**3**)	8
**Figure S11.** HMQC spectrum of (*E*)-4-(4'-benzyloxy-3'-methoxyphenyl)but-3-en-2-yl propionate (**3**)	9
**Figure S12.** HMBC spectrum of (*E*)-4-(4'-benzyloxy-3'-methoxyphenyl)but-3-en-2-yl propionate (**3**)	9
**Figure S13**. ^{1}H NMR spectrum of (*S*)-4-(4'-hydroxy-3'-methoxyphenyl)butan-2-ol ((*S*)-zingerol)	10
**Figure S14.** ^{13}C NMR spectrum of (*S*)-4-(4'-hydroxy-3'-methoxyphenyl)butan-2-ol ((*S*)-zingerol)	10
**Figure S15.** IR spectrum of (*E*)-4-(4'-benzyloxy-3'-methoxyphenyl)but-3-en-2-yl acetate (**2**)	11
**Figure S16.** IR spectrum of (*E*)-4-(4'-benzyloxy-3'-methoxyphenyl)but-3-en-2-yl propionate (**3**)	11
**Figure S17.** HR-MS spectrum of (*E*)-4-(4'-benzyloxy-3'-methoxyphenyl)but-3-en-2-yl acetate (**2**)	12
**Figure S18.** HR-MS spectrum of (*E*)-4-(4'-benzyloxy-3'-methoxyphenyl)but-3-en-2-yl propionate (**3**)	12
**Figure S19.** Chiral HPLC chromatogram of the racemic mixture of allylic alcohol (*E*)-4-(4′-benzyloxy-3′-methoxyphenyl)but-3-en-2-ol (**1**)	13
**Figure S20.** Chiral HPLC chromatogram of the racemic mixture of (*E*)-4-(4'-benzyloxy-3'-methoxyphenyl)but-3-en-2-yl propionate (**3**)	13
**Figure S21.** Chiral HPLC chromatogram of (-)-(*S*,*E*)-4-(4′-Benzyloxy-3′-methoxyphenyl)but-3-en-2-ol ((***S***)-**1**)	14
**Figure S22.** Chiral HPLC chromatogram of (+)-(*R*,*E*)-4-(4'-(Benzyloxy)-3'-methoxyphenyl)but-3-en-2-yl propionate ((***R***)-**3**)	14
**Figure S23.** Chiral HPLC chromatogram of (+)-(*R*, *E*)-4-(4′-Benzyloxy-3′-methoxyphenyl)but-3-en-2-ol ((***R***)-**1**)	15
**Figure S24.** Chiral HPLC chromatogram of (-)-*cis*-(4S,5*S*,6*R*)-4-(4′-Hydroxy-3′-methoxyphenyl)-5-(1-iodoethyl)dihydrofuran-2-one ((4*S*,5*S*,6*R*)-**7a**)	15
**Figure S25.** Chiral HPLC chromatogram of (+)-*cis*-(4*R*,5*R*,6*S*)-4-(4′-Hydroxy-3′-methoxyphenyl)-5-(1-iodoethyl)dihydrofuran-2-one ((4*R*,5*R*,6*S*)-**7a**)	16
**Figure S26.** Chiral HPLC chromatogram of (-)-*trans*-(4*S*,5*R*,6*S*)-4-(4′-Hydroxy-3′-methoxyphenyl)-5-(1-iodoethyl)dihydrofuran-2-one ((4*S*,5*R*,6*S*)-**7b**)	16
**Figure S27.** Chiral HPLC chromatogram of (+)-*trans*-(4*R*,5*S*,6*R*)-4-(4′-Hydroxy-3′-methoxyphenyl)-5-(1-iodoethyl)dihydrofuran-2-one ((4*R*,5*S*,6*R*)-**7b**)	17
**Figure S28.** Effect of the tested compound on cell cycle distribution. Representative DNA content histograms of control cells (**A**) and cells treated with the tested compound at concentrations of 0.5 μg/mL (**B**), 1 μg/mL (**C**), 2 μg/mL	17

(**D**), 5 μg/mL (**E**), 10 μg/mL (**F**), and 20 μg/mL (**G**). The X-axis represents propidium iodide (PI) fluorescence intensity, corresponding to cellular DNA content, while the Y-axis represents the number of recorded events.	
**Table S1.** Summary of absolute configuration assignments and analytical data for enantiomerically enriched compounds **1-7.**	18-19

NMR SPECTRA:

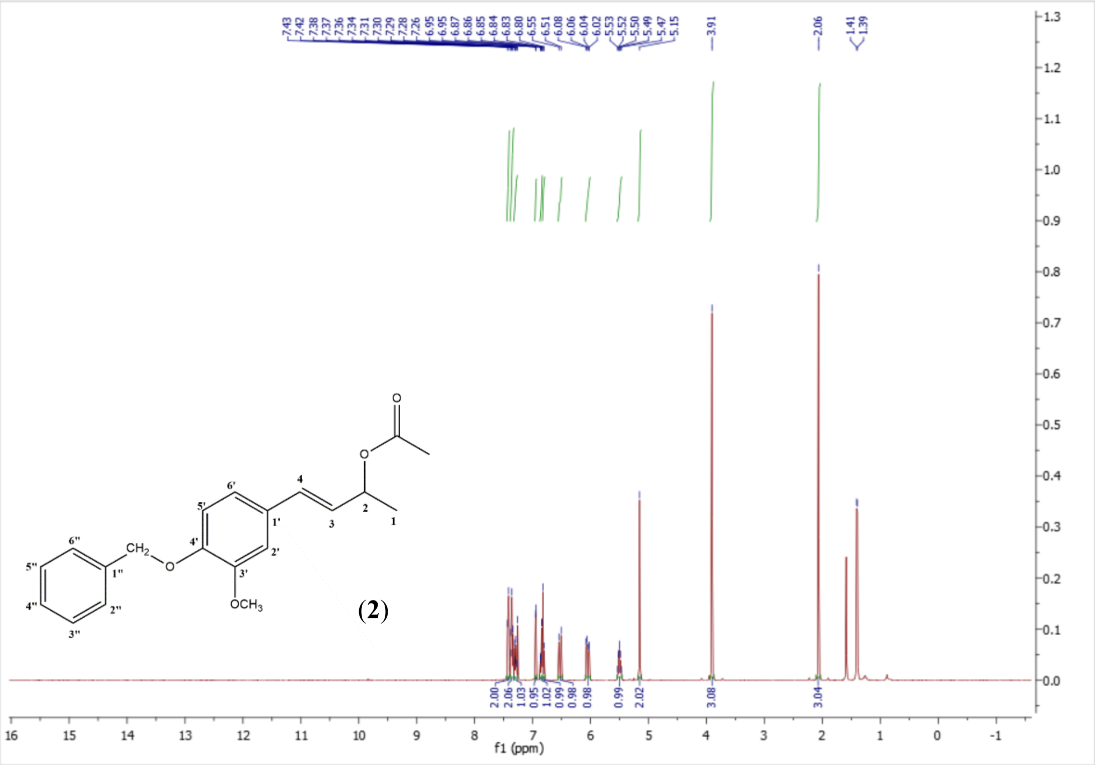

Figure S1. ^{1}H NMR spectrum of (*E*)-4-(4'-benzyloxy-3'-methoxyphenyl)but-3-en-2-yl acetate (**2**)

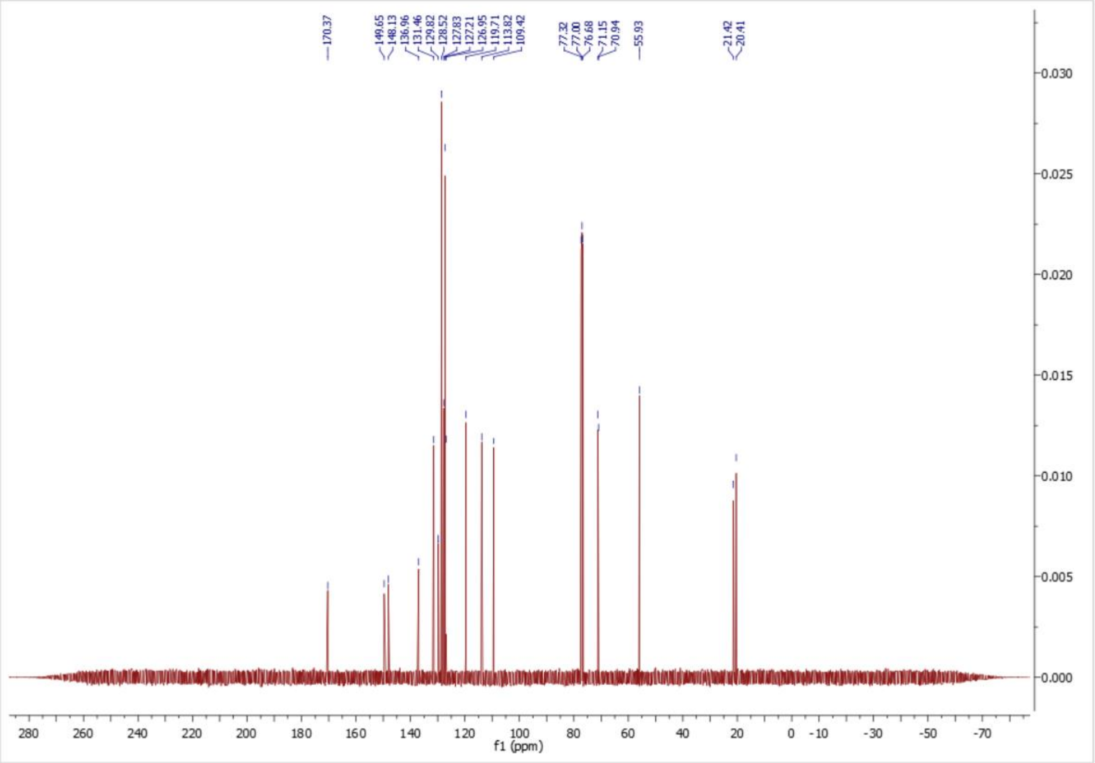

Figure S2. ^{13}C NMR spectrum of (*E*)-4-(4'-benzyloxy-3'-methoxyphenyl)but-3-en-2-yl acetate (**2**)

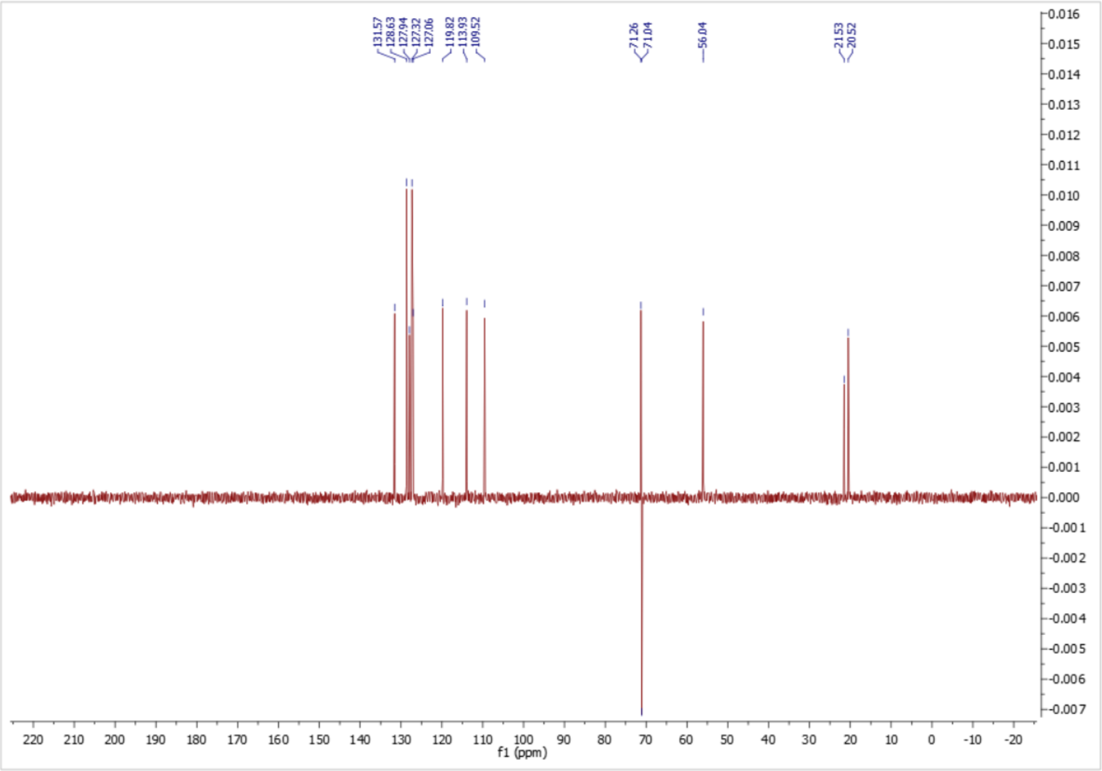

Figure S3. DEPT 135 NMR spectrum of (*E*)-4-(4'-benzyloxy-3'-methoxyphenyl)but-3-en-2-yl acetate (**2**)

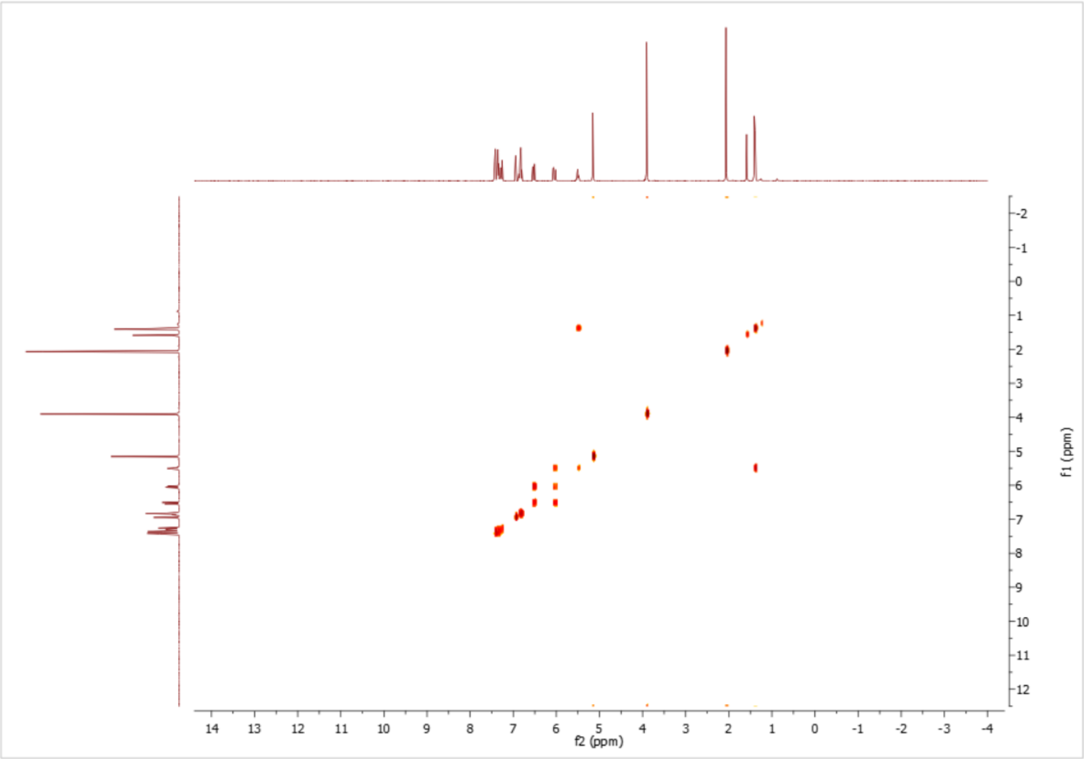

Figure S4. COSY spectrum of (*E*)-4-(4'-benzyloxy-3'-methoxyphenyl)but-3-en-2-yl acetate (**2**)

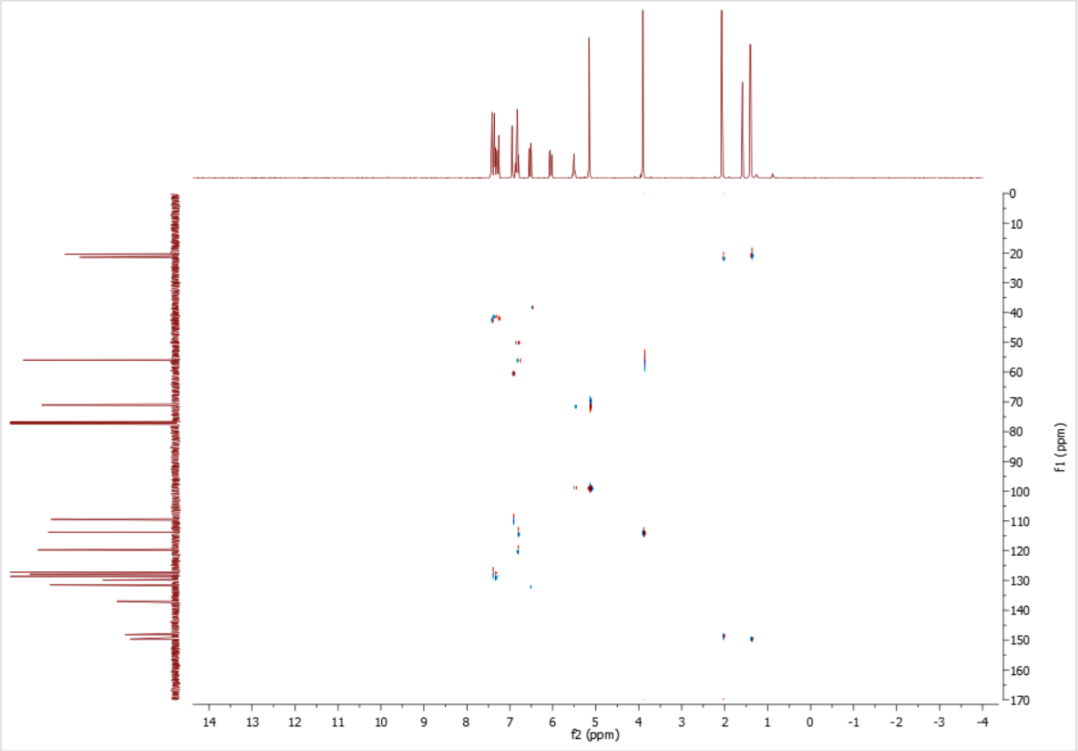

Figure S5. HMQC spectrum of (*E*)-4-(4’-benzyloxy-3’-methoxyphenyl)but-3-en-2-yl acetate (**2**)

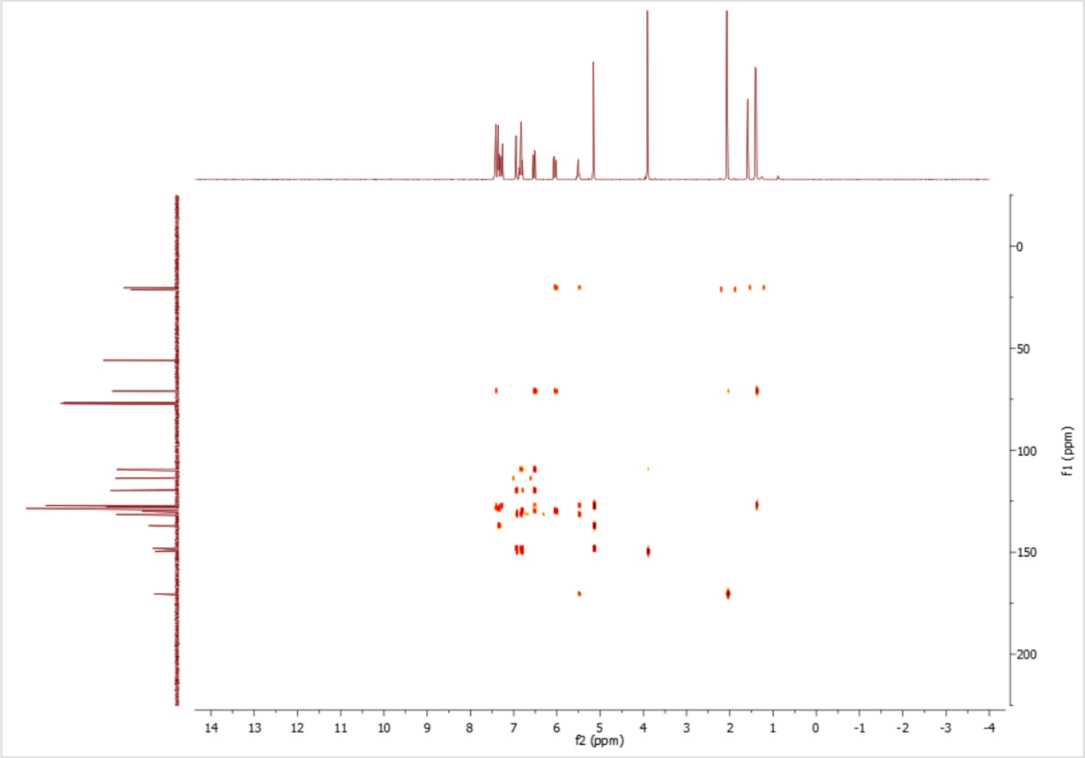

Figure S6. HMBC spectrum of (*E*)-4-(4’-benzyloxy-3’-methoxyphenyl)but-3-en-2-yl acetate (**2**)

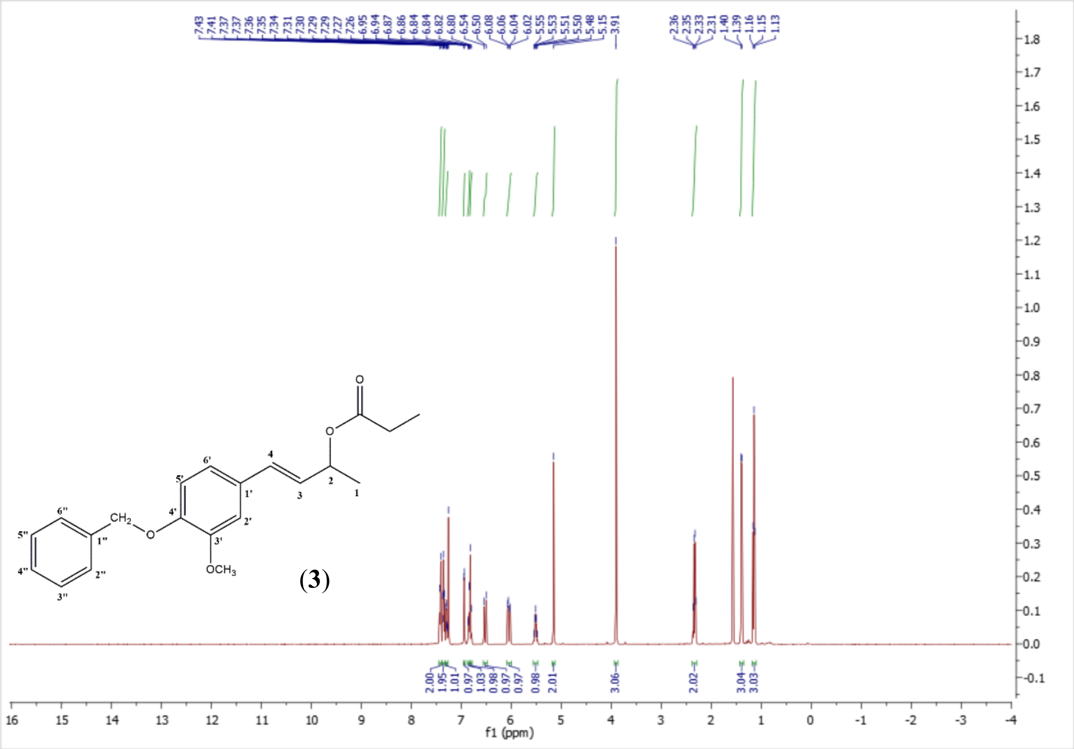

Figure S7. ^{1}H NMR spectrum of (*E*)-4-(4'-benzyloxy-3'-methoxyphenyl)but-3-en-2-yl propionate (**3**)

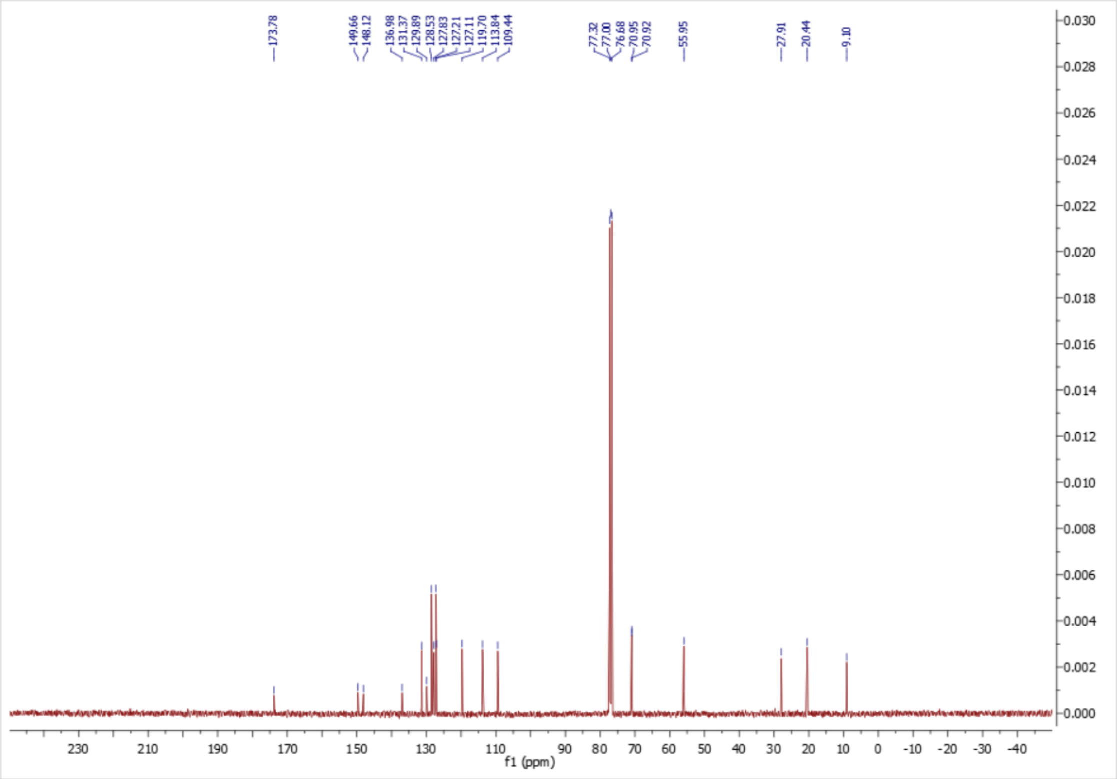

Figure S8. ^{13}C NMR spectrum of (*E*)-4-(4'-benzyloxy-3'-methoxyphenyl)but-3-en-2-yl propionate (**3**)

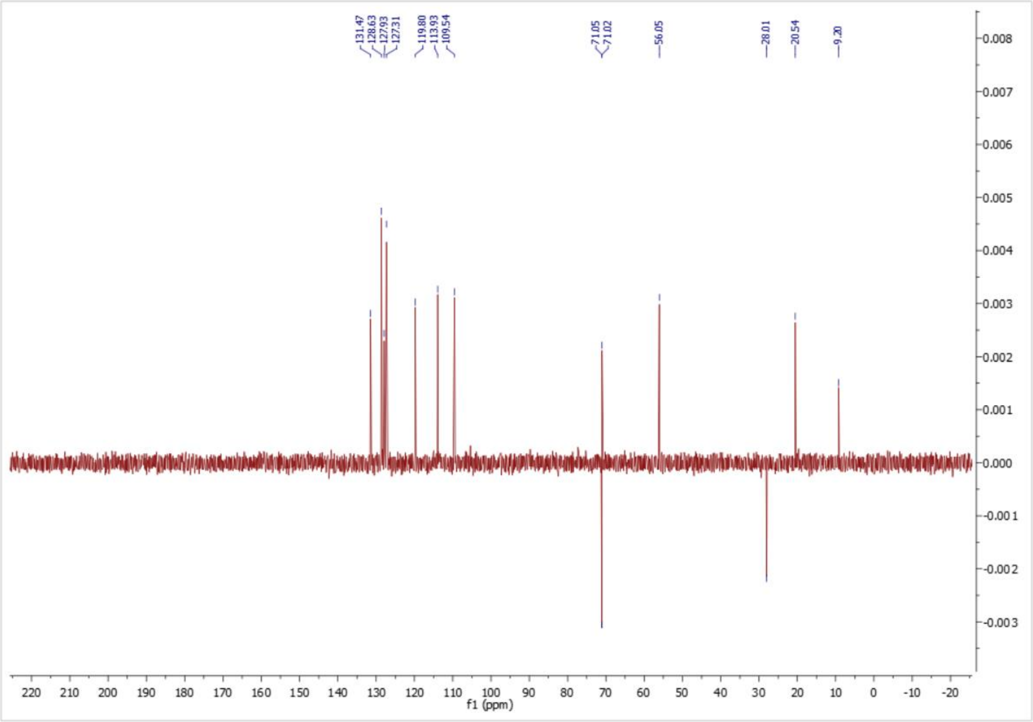

Figure S9. DEPT 135 NMR spectrum of (*E*)-4-(4'-benzyloxy-3'-methoxyphenyl)but-3-en-2-yl propionate (**3**)

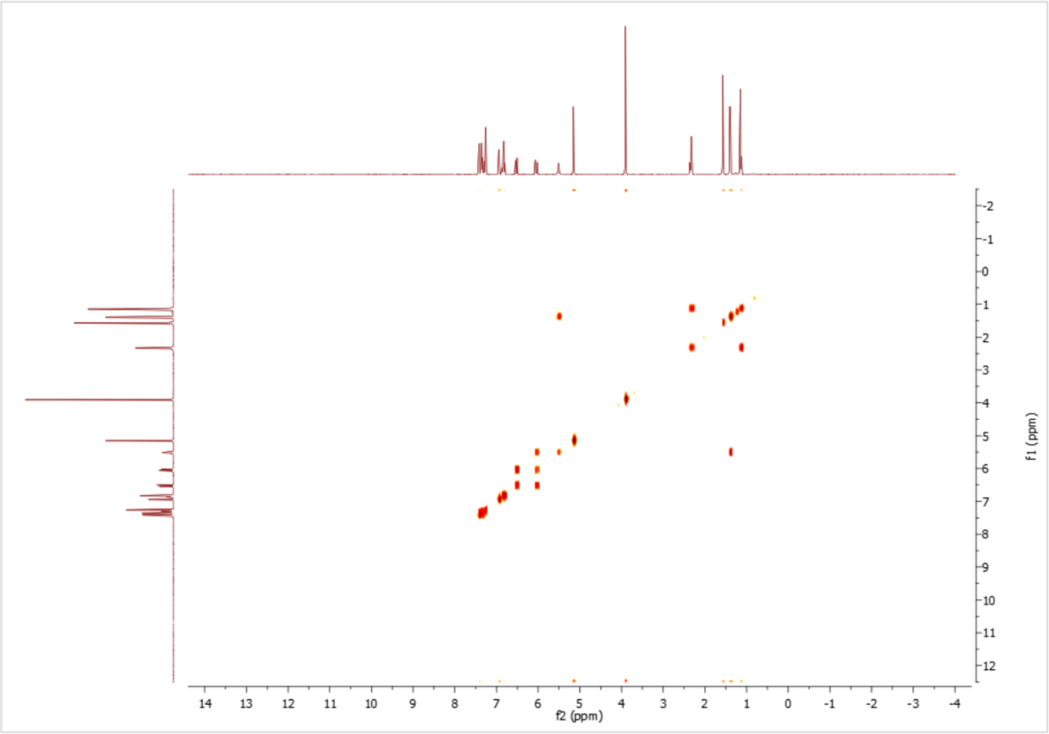

Figure S10. COSY spectrum of (*E*)-4-(4'-benzyloxy-3'-methoxyphenyl)but-3-en-2-yl propionate (**3**)

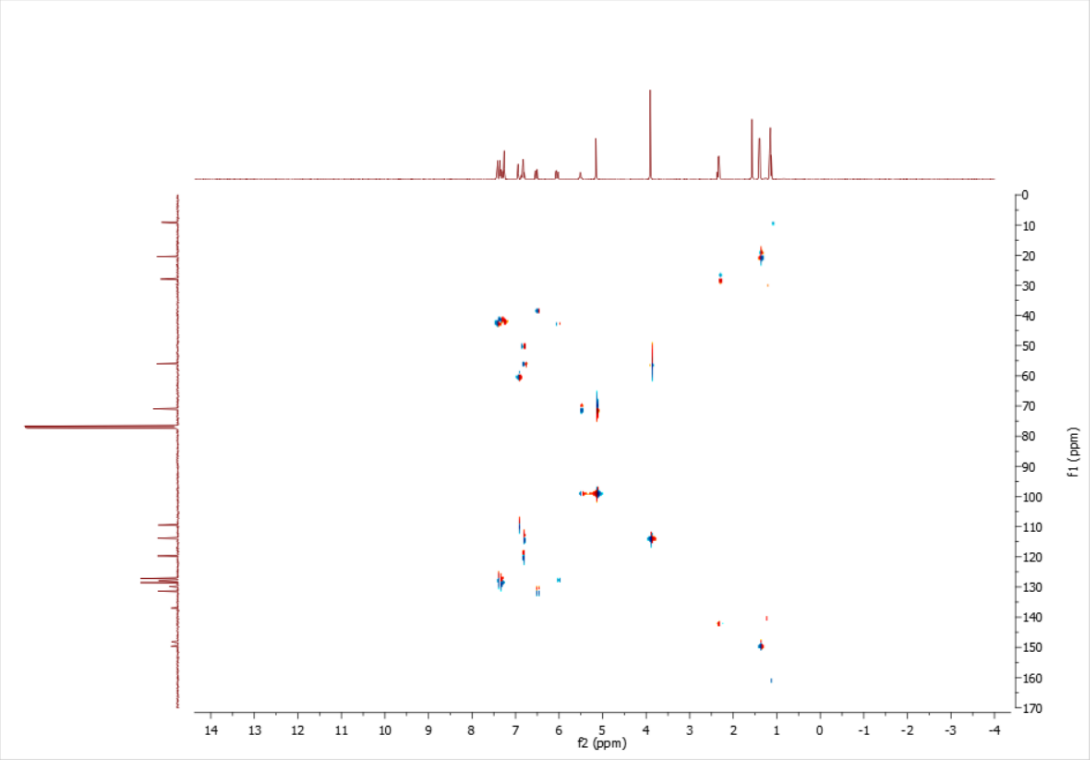

Figure S11. HMQC spectrum of (*E*)-4-(4’-benzyloxy-3’-methoxyphenyl)but-3-en-2-yl propionate (**3**)

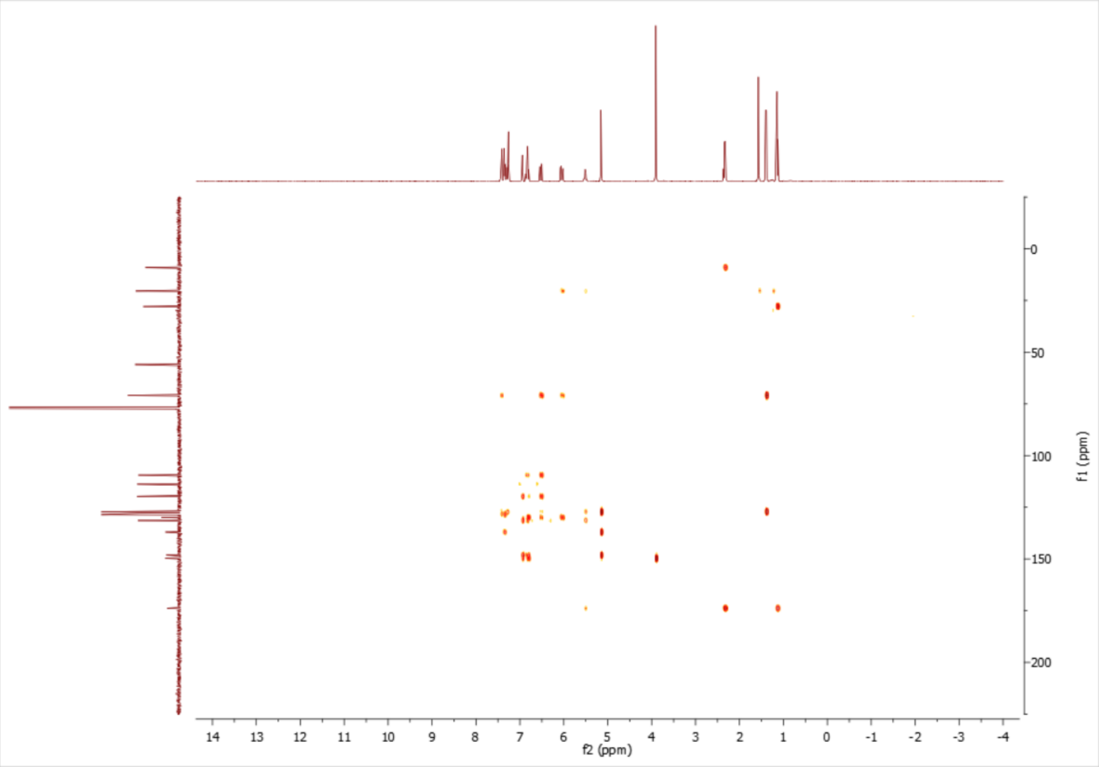

Figure S12. HMBC spectrum of (*E*)-4-(4’-benzyloxy-3’-methoxyphenyl)but-3-en-2-yl propionate (**3**)

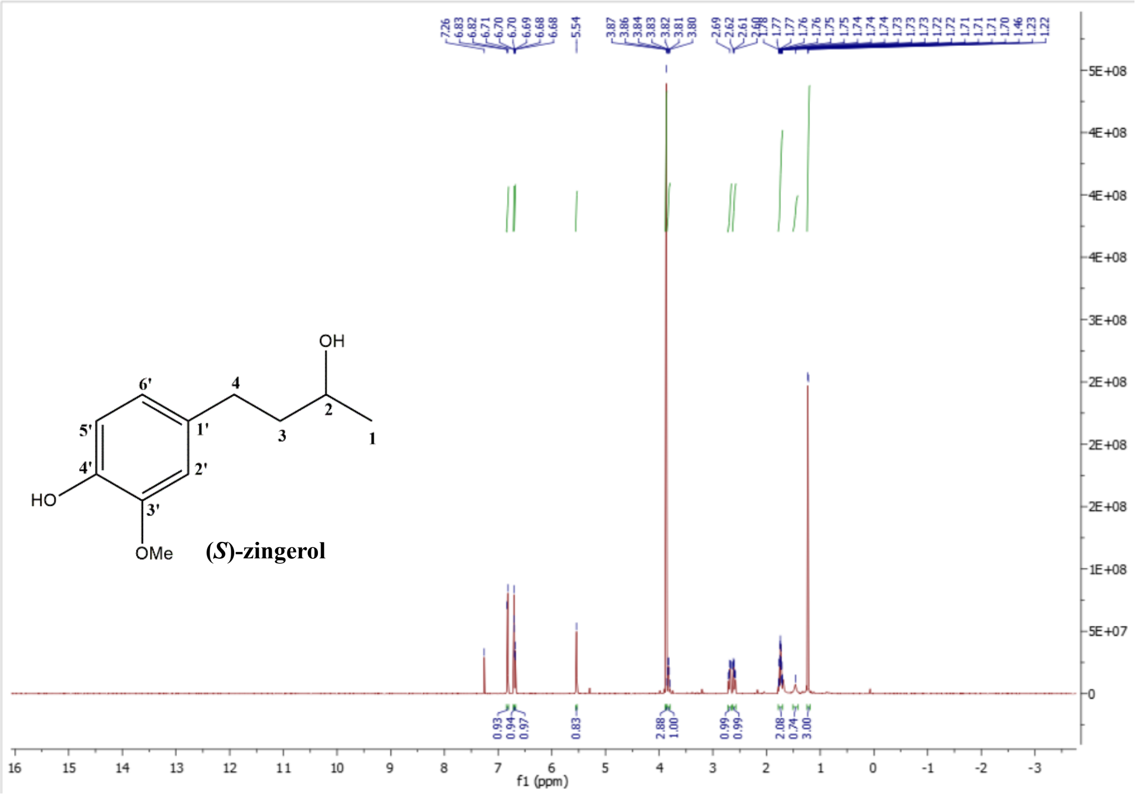

Figure S13. ^{1}H NMR spectrum of (*S*)-4-(4'-hydroxy-3'-methoxyphenyl)butan-2-ol ((***S*)-zingerol**)

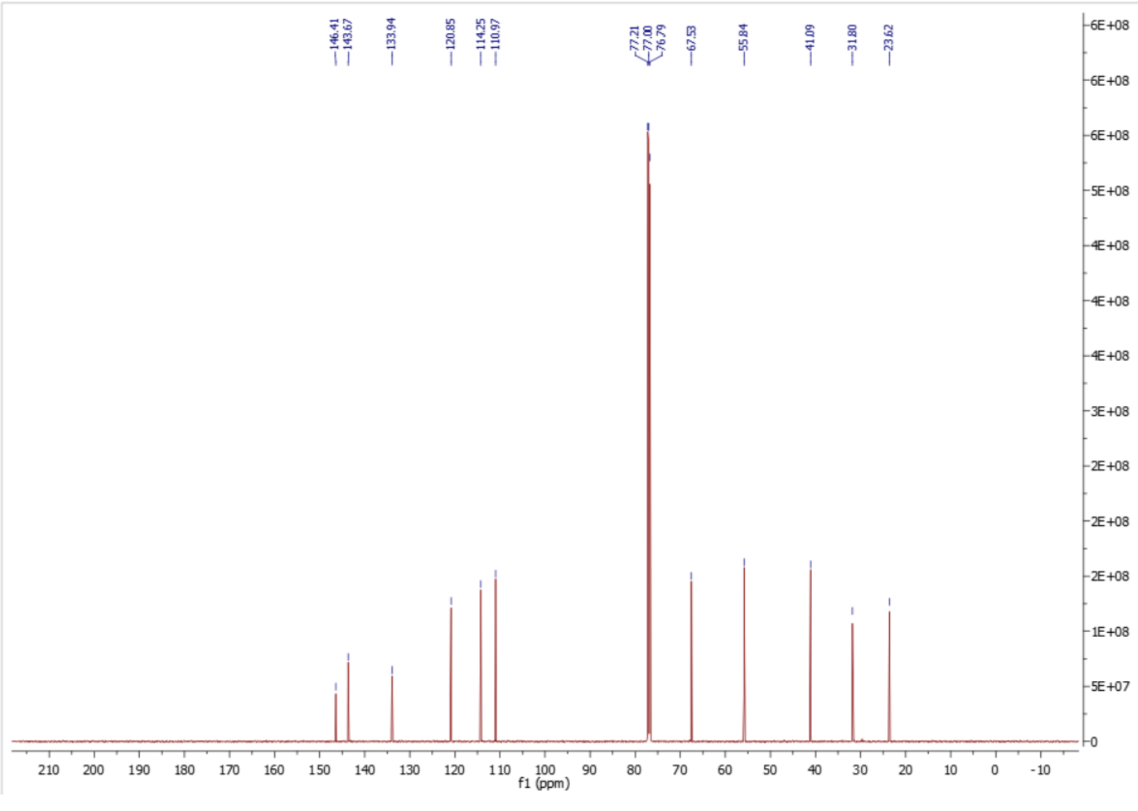

Figure S14. ^{13}C NMR spectrum of (*S*)-4-(4'-hydroxy-3'-methoxyphenyl)butan-2-ol ((***S*)-zingerol**)

IR SPECTRA:

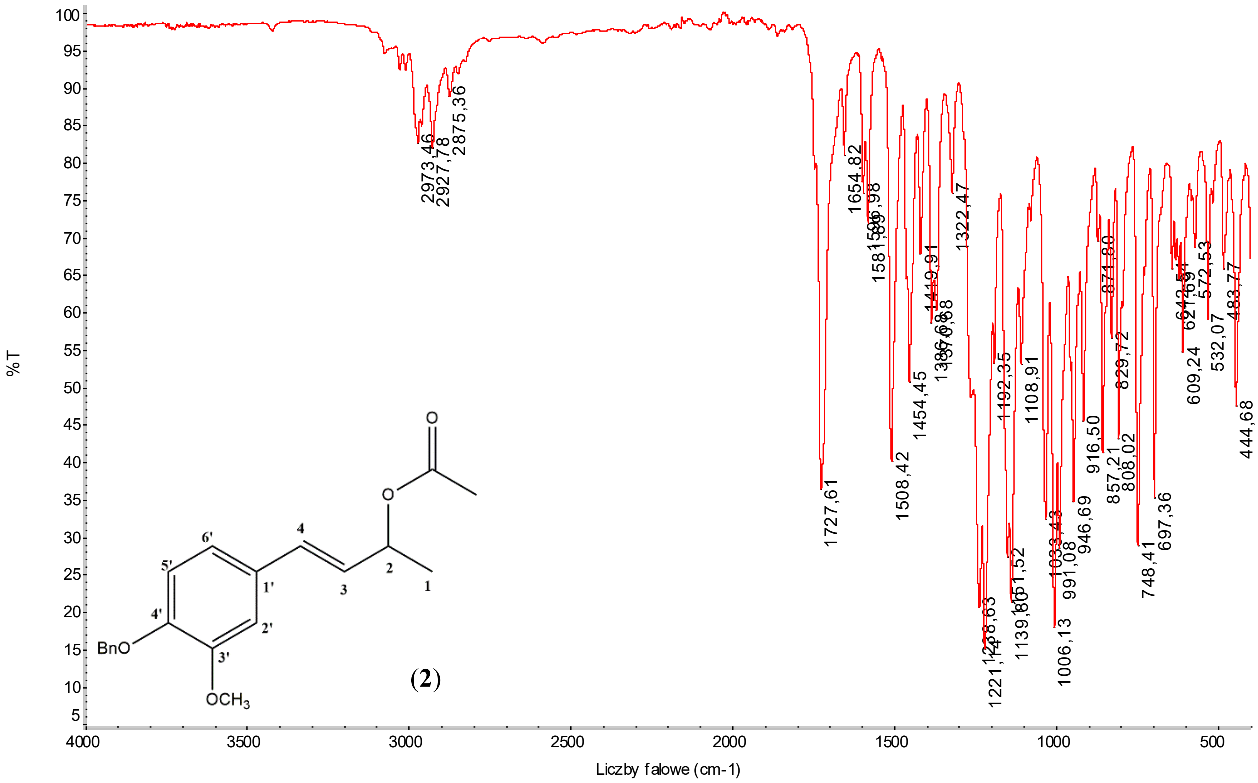

Figure S15. IR spectrum of (*E*)-4-(4'-benzyloxy-3'-methoxyphenyl)but-3-en-2-yl acetate (**2**)

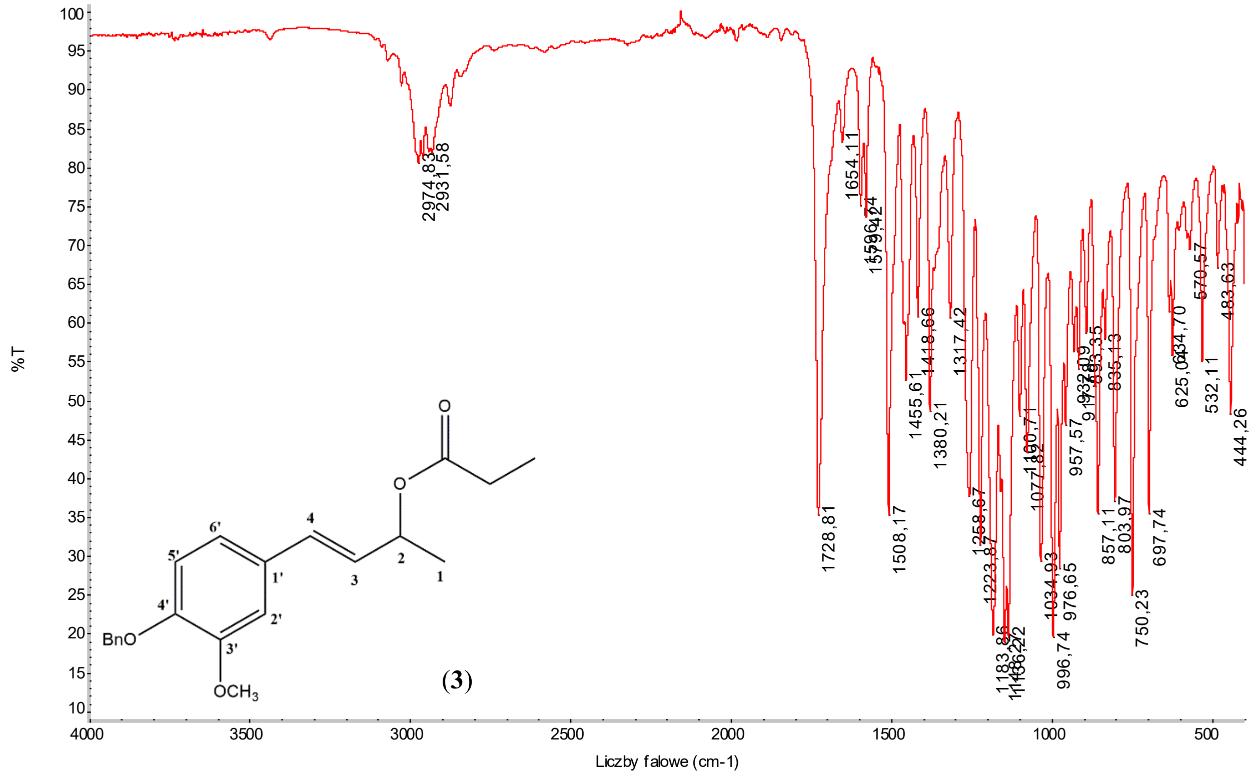

Figure S16. IR spectrum of (*E*)-4-(4'-benzyloxy-3'-methoxyphenyl)but-3-en-2-yl propionate (**3**)

HR-MS SPECTRA:

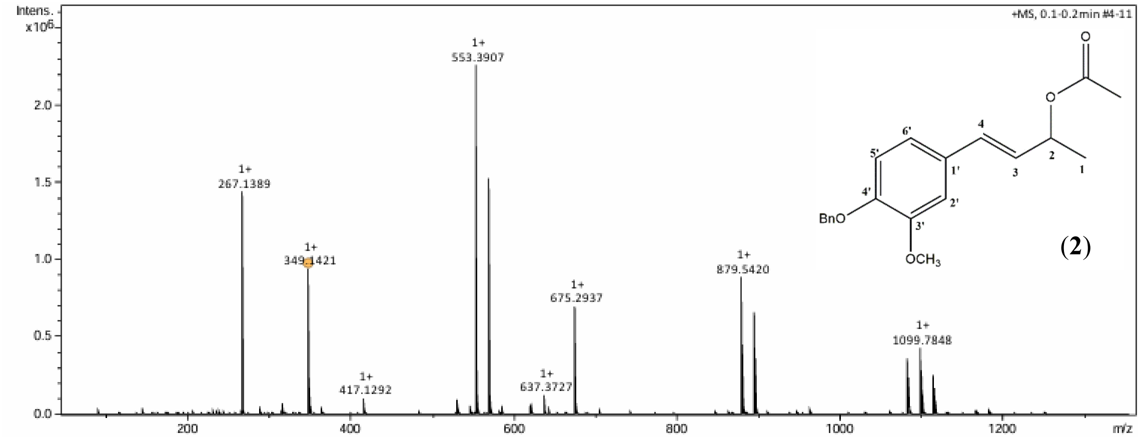

Wyznaczone wzory sumaryczne jonu:

Meas. m/z	#	Ion Formula	m/z	err [ppm]	mSigma	# Sigma	Score	rdb	e— Conf	N-Rule
349.1421	1	**C20H22NaO4**	349.1410	**-2.9**	20.8	1	100.00	9.5	even	ok

Figure S17. HR-MS spectrum of (*E*)-4-(4'-benzyloxy-3'-methoxyphenyl)but-3-en-2-yl acetate (**2**)

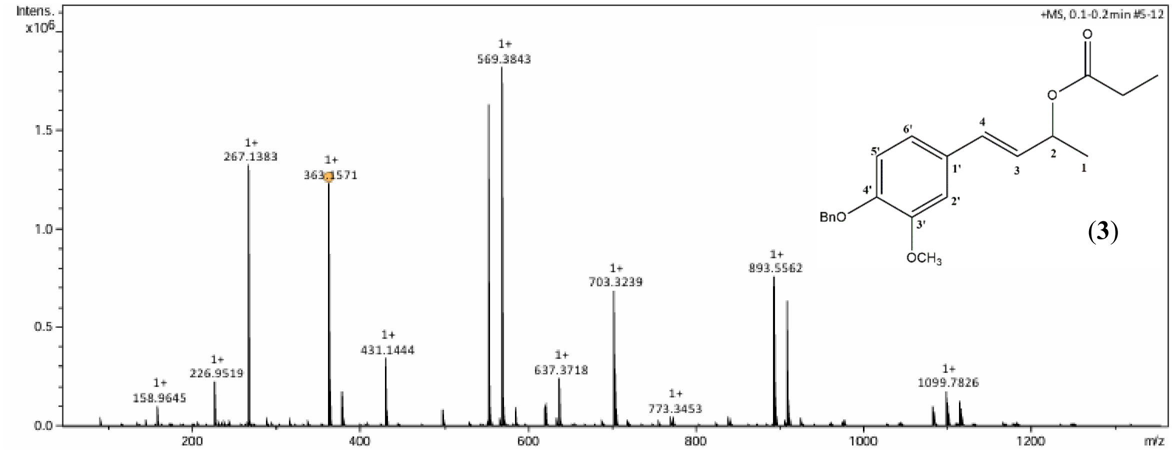

Wyznaczone wzory sumaryczne jonu:

Meas. m/z	#	Ion Formula	m/z	err [ppm]	mSigma	# Sigma	Score	rdb	e— Conf	N-Rule
363.1571	1	**C21H24NaO4**	363.1567	**1.1**	21.6	1	100.00	9.5	even	ok

Figure S18. HR-MS spectrum of (*E*)-4-(4'-benzyloxy-3'-methoxyphenyl)but-3-en-2-yl propionate (**3**)

CHIRAL HPLC SPECTRA:

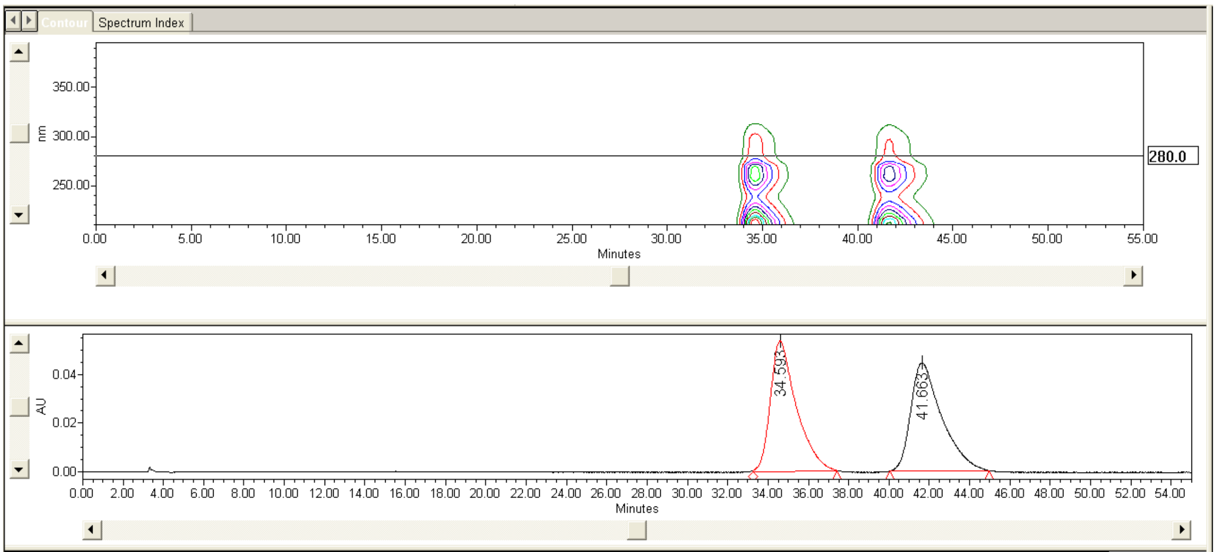

	Name	Retention Time (min)	Purity1 Angle	Purity1 Threshold	PDA Match1 Spect. Name	PDA Match1 Angle	PDA Match1 Threshold	PDA Match1 Lib. Name	Area (µV*sec)	% Area	Height (µV)	Int Type	Amount	Units	Peak Type	Peak Codes
1		34.593							4798657	50.40	53709	VB			Unknown	
2		41.663							4722743	49.60	44397	BB			Unknown	

Figure S19. Chiral HPLC chromatogram of the racemic mixture of allylic alcohol (*E*)-4-(4′-benzyloxy-3′-methoxyphenyl)but-3-en-2-ol (**1**)

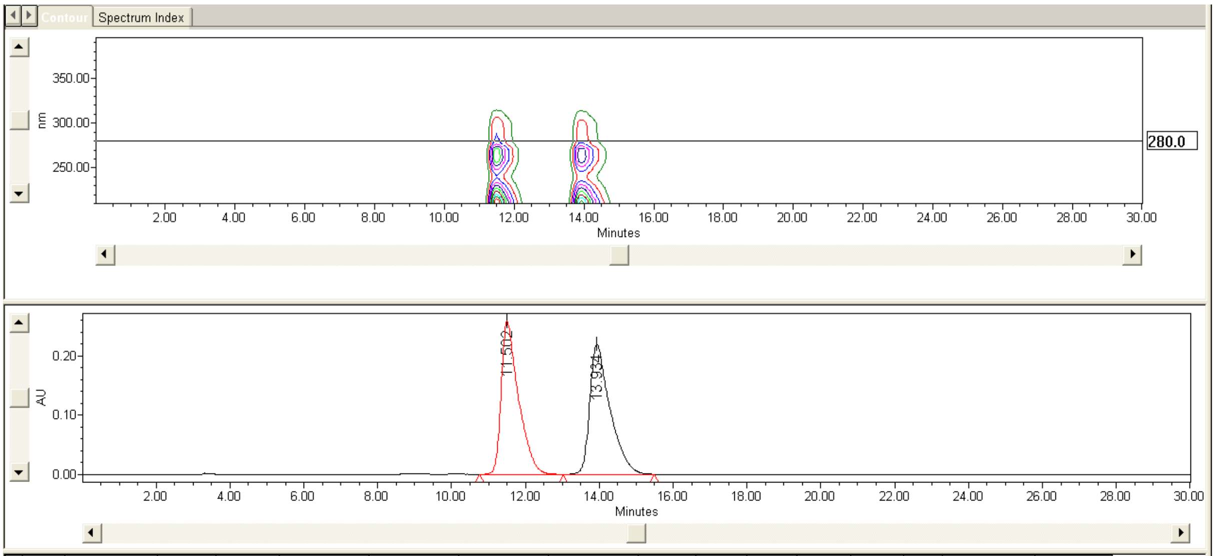

	Name	Retention Time (min)	Purity1 Angle	Purity1 Threshold	PDA Match1 Spect. Name	PDA Match1 Angle	PDA Match1 Threshold	PDA Match1 Lib. Name	Area (µV*sec)	% Area	Height (µV)	Int Type	Amount	Units	Peak Type	Peak Codes
1		11.502							8365518	50.02	258837	BB			Unknown	
2		13.934							8357370	49.98	219332	BB			Unknown	

Figure S20. Chiral HPLC chromatogram of the racemic mixture of (*E*)-4-(4’-benzyloxy-3’-methoxyphenyl)but-3-en-2-yl propionate (**3**)

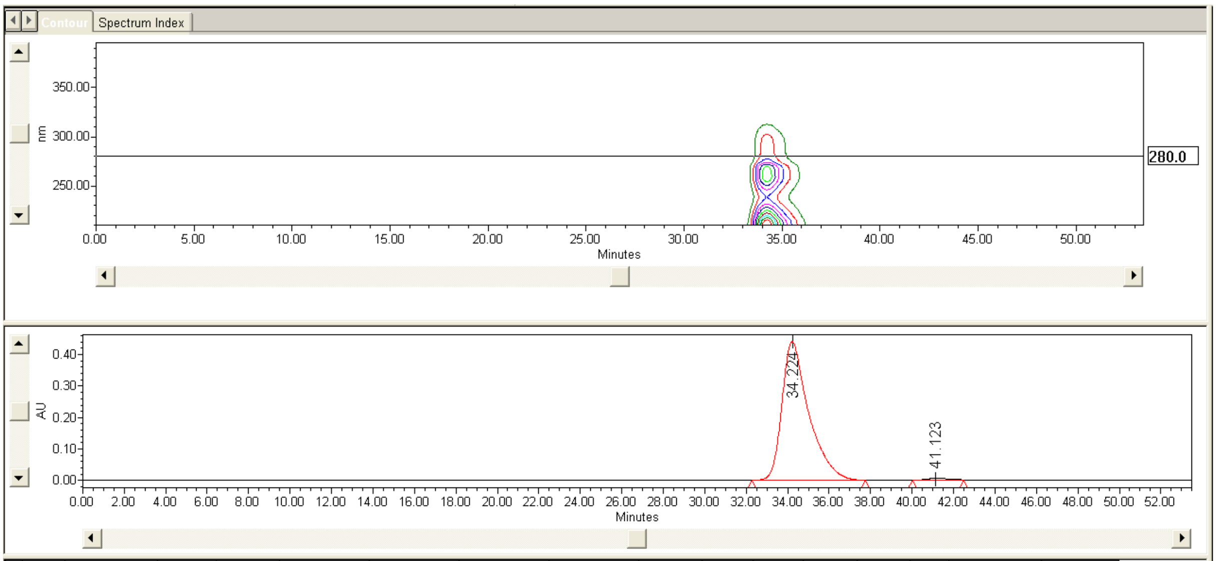

	Name	Retention Time (min)	Purity1 Angle	Purity1 Threshold	PDA Match1 Spect. Name	PDA Match1 Angle	PDA Match1 Threshold	PDA Match1 Lib. Name	Area (µV*sec)	% Area	Height (µV)	Int Type	Amount	Units	Peak Type	Peak Codes
1		34.224							39737908	98.97	441139	BB			Unknown	
2		41.123							412254	1.03	5524	BB			Unknown	

3D Channels / 2D Channels / Peaks

Figure S21. Chiral HPLC chromatogram of (-)-(*S*,*E*)-4-(4′-Benzyloxy-3′-methoxyphenyl)but-3-en-2-ol ((***S***)-**1**)

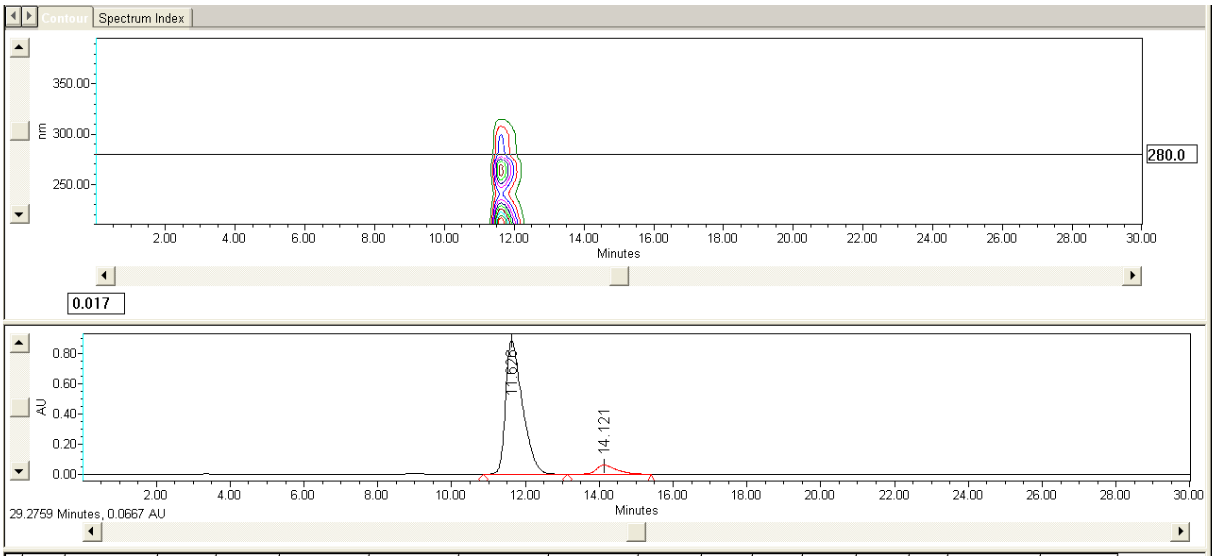

	Name	Retention Time (min)	Purity1 Angle	Purity1 Threshold	PDA Match1 Spect. Name	PDA Match1 Angle	PDA Match1 Threshold	PDA Match1 Lib. Name	Area (µV*sec)	% Area	Height (µV)	Int Type	Amount	Units	Peak Type	Peak Codes
1		11.626							28022547	92.65	888592	VV			Unknown	
2		14.121							2221696	7.35	59693	VB			Unknown	

3D Channels / 2D Channels / Peaks

Figure S22. Chiral HPLC chromatogram of (+)-(*R*,*E*)-4-(4’-(Benzyloxy)-3’-methoxyphenyl)but-3-en-2-yl propionate ((***R***)-**3**)

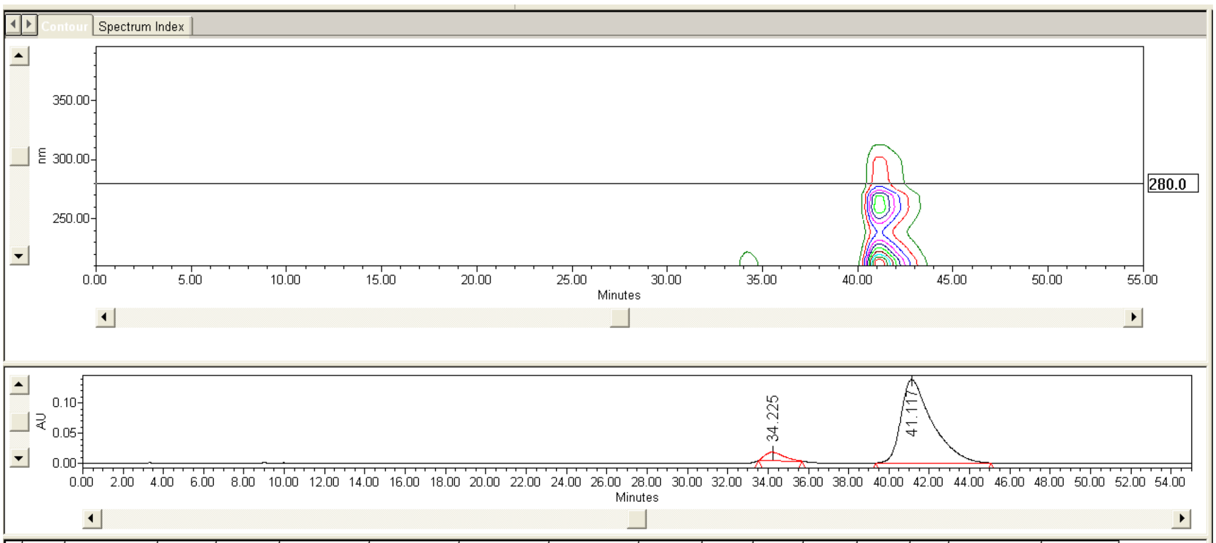

	Name	Retention Time (min)	Purity1 Angle	Purity1 Threshold	PDA Match1 Spect. Name	PDA Match1 Angle	PDA Match1 Threshold	PDA Match1 Lib. Name	Area (µV*sec)	% Area	Height (µV)	Int Type	Amount	Units	Peak Type	Peak Codes
1		34.225							905671	5.74	14027	bb			Unknown	
2		41.117							14878953	94.26	138311	BB			Unknown	

3D Channels | 2D Channels | Peaks

Figure S23. Chiral HPLC chromatogram of (+)-(*R*, *E*)-4-(4′-Benzyloxy-3′-methoxyphenyl)but-3-en-2-ol ((***R***)-**1**)

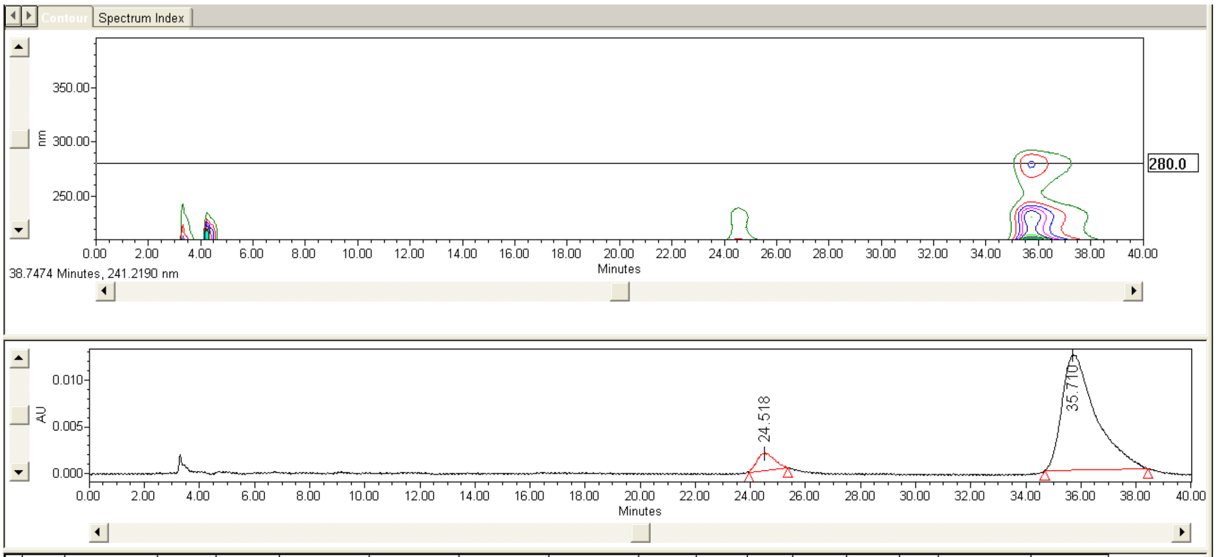

	Name	Retention Time (min)	Purity1 Angle	Purity1 Threshold	PDA Match1 Spect. Name	PDA Match1 Angle	PDA Match1 Threshold	PDA Match1 Lib. Name	Area (µV*sec)	% Area	Height (µV)	Int Type	Amount	Units	Peak Type	Peak Codes
1		24.518							79700	7.08	1860	Bb			Unknown	
2		35.710							1046667	92.92	12289	Bb			Unknown	

3D Channels | 2D Channels | Peaks

Figure S24. Chiral HPLC chromatogram of (-)-*cis*-(4S,5*S*,6*R*)-4-(4′-Hydroxy-3′-methoxyphenyl)-5-(1-iodoethyl)dihydrofuran-2-one ((4*S*,5*S*,6*R*)-**7a**)

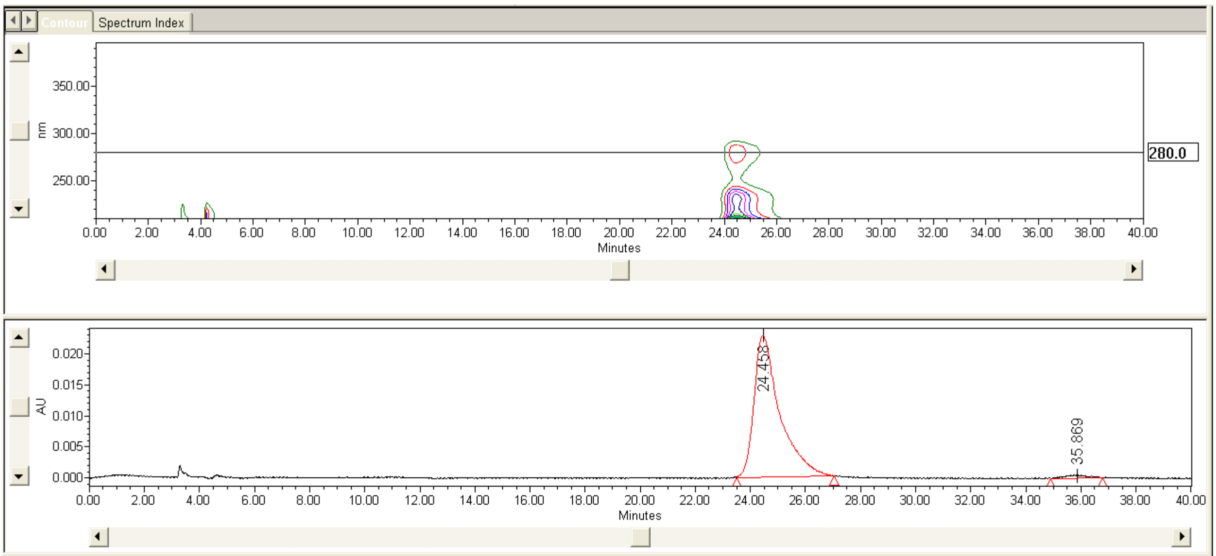

Name	Retention Time (min)	Purity1 Angle	Purity1 Threshold	PDA Match1 Spect. Name	PDA Match1 Angle	PDA Match1 Threshold	PDA Match1 Lib. Name	Area (µV*sec)	% Area	Height (µV)	Int Type	Amount	Units	Peak Type	Peak Codes
1	24.458							1482739	98.33	22841	Bb			Unknown	
2	35.869							25130	1.67	449	bb			Unknown	

3D Channels / 2D Channels / Peaks

Figure S25. Chiral HPLC chromatogram of (+)-*cis*-(4*R*,5*R*,6*S*)-4-(4′-Hydroxy-3′-methoxyphenyl)-5-(1-iodoethyl)dihydrofuran-2-one ((4*R*,5*R*,6*S*)-**7a**)

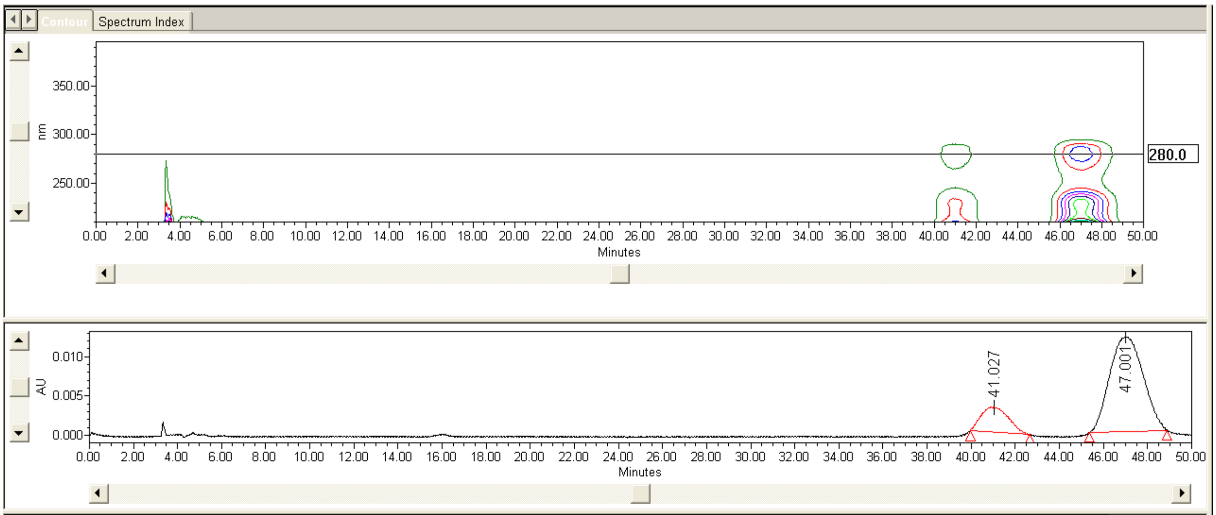

Name	Retention Time (min)	Purity1 Angle	Purity1 Threshold	PDA Match1 Spect. Name	PDA Match1 Angle	PDA Match1 Threshold	PDA Match1 Lib. Name	Area (µV*sec)	% Area	Height (µV)	Int Type	Amount	Units	Peak Type	Peak Codes
1	41.027							277243	17.95	3234	Bb			Unknown	
2	47.001							1267275	82.05	12053	BB			Unknown	

3D Channels / 2D Channels / Peaks

Figure S26. Chiral HPLC chromatogram of (-)-*trans*-(4*S*,5*R*,6*S*)-4-(4′-Hydroxy-3′-methoxyphenyl)-5-(1-iodoethyl)dihydrofuran-2-one ((4*S*,5*R*,6*S*)-**7b**)

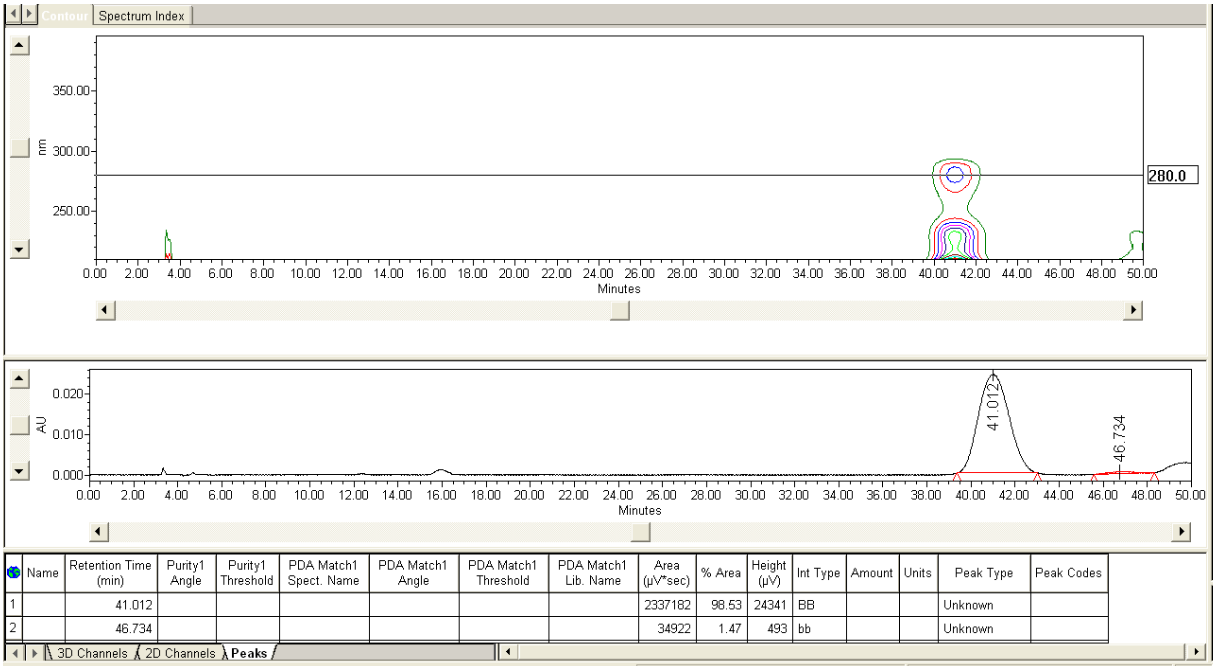

	Name	Retention Time (min)	Purity1 Angle	Purity1 Threshold	PDA Match1 Spect. Name	PDA Match1 Angle	PDA Match1 Threshold	PDA Match1 Lib. Name	Area (µV*sec)	% Area	Height (µV)	Int Type	Amount	Units	Peak Type	Peak Codes
1		41.012							2337182	98.53	24341	BB			Unknown	
2		46.734							34922	1.47	493	bb			Unknown	

Figure S27. Chiral HPLC chromatogram of (+)-*trans*-(4*R*,5*S*,6*R*)-4-(4′-Hydroxy-3′-methoxyphenyl)-5-(1-iodoethyl)dihydrofuran-2-one ((4*R*,5*S*,6*R*)-**7b**)

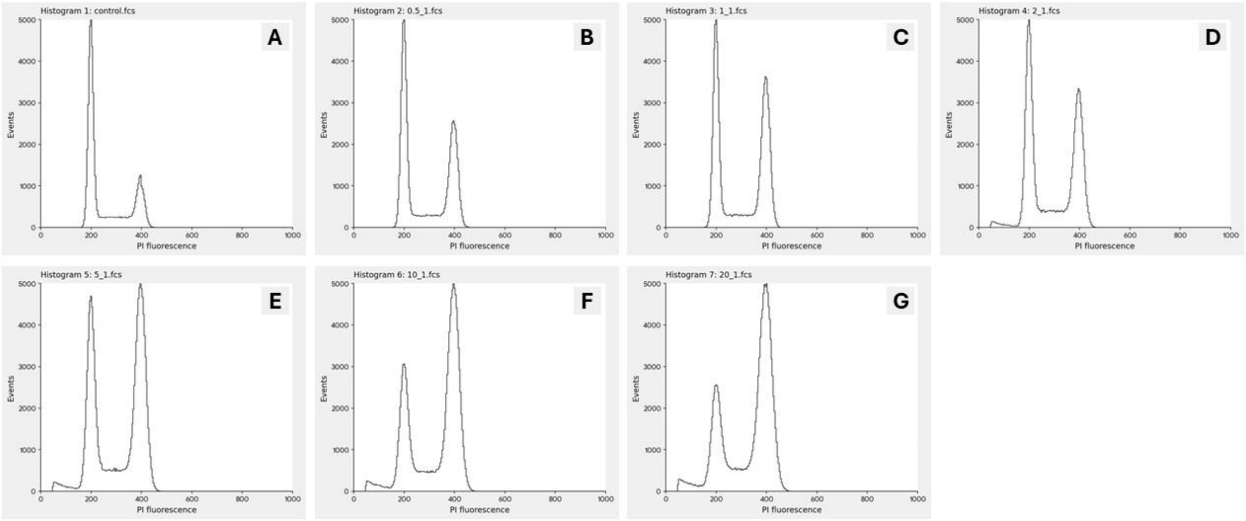

Figure S28. Effect of the tested compound on cell cycle distribution. Representative DNA content histograms of control cells (**A**) and cells treated with the tested compound at concentrations of 0.5 µg/mL (**B**), 1 µg/mL (**C**), 2 µg/mL (**D**), 5 µg/mL (**E**), 10 µg/mL (**F**), and 20 µg/mL (**G**). The X-axis represents propidium iodide (PI) fluorescence intensity, corresponding to cellular DNA content, while the Y-axis represents the number of recorded events.

Table S1. Summary of absolute configuration assignments and analytical data for enantiomerically enriched compounds **1-7**.

Compound	Assigned configuration	ee [%]	Chiral HPLC retention time [min]	Specific rotation [$[\alpha]_D^{20}$]	Basis for assignment
1	*S*	98	34.2	-30.6 (*c* 1.0, CH_2Cl_2)	Chemical correlation with known (*S*)-zingerol obtained by catalytic hydrogenation and comparison of specific rotation with literature data;
1	*R*	89	41.1	+28.8 (*c* 1.05, CH_2Cl_2)	Absolute configuration of the faster-reacting enantiomer in the transesterification was assigned as opposite to that of the slower-reacting (*S*)-1 enantiomer
3	*R*	85	11.6	+21.9 (*c* 1.05, CH_2Cl_2)	Stereochemical correlation with the assigned (*R*)-**1**
4	*R*	76	9.1	-6.5 (*c* 1.0, CH_2Cl_2)	Stereochemical correlation with (*R*)-**1;** retention of configuration during highly stereoselective Claisen-Johnson rearrangement
4	*S*	95	9.6	+7.8 (*c* 1.0, CH_2Cl_2)	Stereochemical correlation with (*S*)-**1**; retention of configuration during highly stereoselective Claisen-Johnson rearrangement
5	*R*	81	28.0	-1.2 (*c* 1.0, CH_2Cl_2)	Stereochemical correlation; retention of configuration after hydrolysis of ester (*R*)-**4**
5	*S*	94	25.9	+1.4 (*c* 1.0, CH_2Cl_2)	Stereochemical correlation; retention of configuration after hydrolysis of ester (*S*)-**4**
6a	(4*S*,5*S*,6*R*)	81	27.6	-3.9 (*c* 1.0, CH_2Cl_2)	Stereochemical correlation with (*R*)-**5** and mechanistic assignments based on a stereospecific iodolactonization pathway; the configuration at C-4 is inherited from the starting acid, while the configurations at C-5 and C-6 are assigned based on an anti-nucleophilic attack on the iodonium intermediate
6b	(4*S*,5*R*,6*S*)	64	38.6	-6.8 (*c* 1.0, CH_2Cl_2)	
6c	(4*S*,5*S*,6*R*)	>99	88.4	+20.1 (*c* 0.7, CH_2Cl_2)	
6a	(4*R*,5*R*,6*S*)	85	19.5	+4.0 (*c* 1.0, CH_2Cl_2)	Stereochemical correlation with (*S*)-**5** and mechanistic assignments based on a stereospecific iodolactonization pathway; the configuration at C-4 is inherited from the starting acid, while the configurations at C-5 and C-6 are assigned based on an anti-nucleophilic attack on the iodonium intermediate
6b	(4*R*,5*S*,6*R*)	74	33.1	+ 9.1 (*c* 1.0, CH_2Cl_2)	
6c		>99	86.4	-20.2 (*c* 1.0, CH_2Cl_2)	

	(4*R*,5*R*,6*S*)				
7a	(4*S*,5*S*,6*R*)	86	35.7	-16.8 (*c* 1.5, CH_2Cl_2)	Stereochemical correlation with (4*S*,5*S*,6*R*)-**6a**; configuration retained during selective debenzylation with BCl_3
7a	(4*R*,5*R*,6*S*)	97	24.5	+18.9 (*c* 1.7, CH_2Cl_2)	Stereochemical correlation with (4*R*,5*R*,6*S*)-**6a**; configuration retained during selective debenzylation with BCl_3
7b	(4*S*,5*R*,6*S*)	64	47.1	-8.9 (*c* 1.3, CH_2Cl_2)	Stereochemical correlation with (4*S*,5*R*,6*S*)-**6b**; configuration retained during selective debenzylation with BCl_3
7b	(4*R*,5*S*,6*R*)	97	41.0	+12.3 (*c* 2.2, CH_2Cl_2).	Stereochemical correlation with (4*R*,5*S*,6*R*)-**6b**; configuration retained during selective debenzylation with BCl_3
